# Rising Threat: Long‐Term Trends in the Incidence and Mortality of Thymic Epithelial Tumor

**DOI:** 10.1002/cam4.70968

**Published:** 2025-05-15

**Authors:** Zishan Chen, Shiwen Liu, Chunting Chen, Jinmang Zhuang, Xinying Xu, Maolin Liu, Fancai Lai, Fei He

**Affiliations:** ^1^ Department of Epidemiology and Health Statistics, Fujian Provincial Key Laboratory of Environment Factors and Cancer, School of Public Health Fujian Medical University Fuzhou China; ^2^ Department of Thoracic Surgery The First Affiliated Hospital of Fujian Medical University Fuzhou China

**Keywords:** incidence, mortality, prognosis, SEER, Thymic epithelial tumor

## Abstract

**Introduction:**

An upward trend in the incidence of thymic epithelial tumors (TETs) has been reported over the past few decades, but because of its rarity, little is currently known about its epidemiological trends. This study examined temporal trends in the incidence and mortality of TETs in the US and explored these trends in population subgroups while investigating important factors that influence their prognosis.

**Methods:**

A retrospective, population‐based study was conducted using nationally representative data from the Surveillance, Epidemiology, and End Results program, and 4979 patients diagnosed with TETs from 2000 to 2020 were evaluated. Associated population data were used to determine age‐adjusted incidence and mortality, and 5‐year TET‐specific mortality (SM). Trends were assessed for the entire cohort, as well as for particular subgroups, including thymoma and thymic carcinoma.

**Results:**

From 2000 to 2020, the overall incidence and mortality of TETs were 2.769 and 1.203 per million person‐years, respectively. Both the age‐adjusted incidence and mortality of TETs increased over the study period, with increases occurring across almost all ethnic groups, histological subtypes, and stages. Multivariate analysis revealed that age, World Health Organization histological type (B1, B2, and B3 thymoma and thymic carcinoma), Masaoka–Koga stage (IIB and III/IV), maximum tumor diameter (5–10 and > 10 cm), surgery, and chemotherapy were independently associated with TET‐SM.

**Conclusions:**

The incidence and mortality of TETs have steadily increased over time, and these trends might be related to the widespread use of computed tomography for lung cancer screening and the increasing number of TETs found incidentally. The study also identified several important factors independently associated with TET‐SM, suggesting that early diagnosis and surgical intervention are critical to achieving good prognoses.

## 1 Introduction

Thymic epithelial tumors (TETs) originate in the thymus, and they are known for their morphological variability and heterogeneity of tumor epithelial cells [1]. TETs, which can include thymoma and thymic carcinoma, are rare tumors. According to the Surveillance, Epidemiology, and End Results (SEER) database of the US National Cancer Institute (NCI), the incidence rate of TETs in the US is 1.3–3.2 per million [2, 3]. The incidence of this disease is currently increasing [4]. According to the World Health Organization (WHO) classification of 2021, TETs can be further divided into type A thymoma, type AB thymoma, type B1 thymoma, type B2 thymoma, type B3 thymoma, and thymic carcinoma [5]. The classification from type A thymoma to thymic carcinoma reflects a transition from indolent to more aggressive types and differences in the 5‐year survival rate, declining from approximately 90% for thymoma to approximately 55% for thymic carcinoma [6].

Engels et al. [2] analyzed cancer incidence data from the SEER database and found that the incidence of TETs in the United States was 1.3–1.5 per million from 1973 to 2006. Using data from the Netherlands Pathology Registry and Cancer Registry databases, De Jong et al. [3] reported that the incidence of TETs from 1994 to 2003 was 3.2 per million, including rates of 2.2–2.6 per million for thymoma and 0.3–0.6 per million for thymic carcinoma. Studies on the incidence rate of TETs in various countries all confirmed is rarity. However, few studies have examined the long‐term incidence trends of TETs, including only one population‐based incidence trend study, and there is a lack of longitudinal time trend studies on mortality [4].

Surgical resection has always been the main treatment for TETs [7, 8]. However, some patients with advanced or metastatic disease cannot undergo resection, and they can only be treated with radiotherapy and chemotherapy. These patients are prone to recurrence, and their prognosis is poor. Previous publications proposed several independent prognostic factors, such as tumor size, tumor stage, and the WHO histological classification. However, because of the indolent clinical course and rarity of TETs, most prognostic factors are considered controversial. Currently, Masaoka–Koga staging [9, 10] and tumor–node–metastasis (TNM) staging [9] are commonly used in clinical practice to determine the extent and prognosis of TETs, but neither includes tumor size as a parameter. There is controversy regarding whether tumor size affects the prognosis of patients with TETs [11]. Some studies identified correlations of tumor size with overall survival (OS) [11, 12, 13] and recurrence‐free survival (RFS) [11, 14, 15, 16]. However, other studies identified no relationships of tumor size with OS [14, 15, 17, 18, 19] or RFS [13, 18].

Currently, little is known about the epidemiological characteristics, incidence, and mortality trends of TETs, which could be attributable to its rarity and multiple subtypes, as well as the complexity of the classification system. In this study, we described the epidemiological trends of TETs and identified prognostic factors. This study used data from the SEER database and used the annual percentage change (APC) to quantify changes over time. This research is important for understanding the pathogenesis of TETs, and the findings can be used in clinical practice to develop screening guidelines.

## 2 Methods

### 2.1 Data Source

The data were obtained from the SEER database established by the NCI, which records the incidence, mortality, and survival data of malignant tumors in various regions of the US [4, 20]. We used the dataset from SEER 17 registries covering the years 2000–2020 to determine the incidence and mortality trends of TETs and perform descriptive analysis. All patients with TETs were identified using ICD‐O‐3 codes (thymoma: 8580/3, 8581/3, 8582/3, 8583/3, 8584/3, 8585/3; thymic carcinoma: 8020/3, 8023/3, 8033/3, 8070/3, 8082/3, 8123/3, 8140/3, 8144/3, 8260/3, 8310/3, 8430/3, 8560/3, 8586/3, 8980/3) and site codes (C37.9), with only the first match selected for each individual. Cases were required to have been histologically or cytologically confirmed. For survival analysis, TETs must have been diagnosed as the first or only malignant tumor to avoid misdiagnosis because of metastasis. Cases diagnosed within 1 month before death and those diagnosed using death certificates or autopsy reports were included in the analysis of incidence and mortality trends, but they were excluded from survival analysis because survival time was measured in months rather than days (Figure S1).

### 2.2 Study Variables

The variables included in this study were year of diagnosis, year of death, age at diagnosis, sex, race, marital status, histological subtype, Masaoka–Koga stage, tumor size, surgery, chemotherapy, radiotherapy, survival status, survival time, and cause of death. Continuous variables were reported as the median and interquartile range, whereas categorical variables were presented as counts (percentages). Because the SEER database provides SEER staging, we divided cases into four stages based on a study by Fernandes et al. [21] that investigated the role of radiation therapy in TETs treatment, namely stage I–IIA (“invasive tumor confined to the gland” or “localized, not otherwise specified”), stage IIB (“adjacent connective tissue”), stage III–IV (“adjacent organs/structures” or “further adjacent extension” or any positive lymph nodes), and unknown (extent of disease not specified), which corresponded to “localized,” “regional,” “distant,” and unknown, respectively, in the SEER database (Table S1).

Multiple imputation by chained equations was used in this study to impute missing values. We assumed that the data were missing at random (MAR), meaning that the probability of missing data depended on observed data rather than unobserved data. Although MAR cannot be verified statistically, it is a reasonable assumption [22]. We performed 50 iterations of five imputations for the missing data.

### 2.3 Statistical Analysis

SEER*Stat 8.4.3 was used to calculate the age‐standardized incidence rate (ASIR) and age‐standardized mortality rate (ASMR) of TETs based on Segi's world standard population, with the rates reported to five decimal places. The joinpoint regression model, which is based on the time characteristics of disease distribution, was employed to establish segmented regressions by dividing the study period into different intervals using several joinpoints. Each segment was fitted and optimized using a continuous linear or logarithmic trend model, permitting a more detailed evaluation of the specific disease changes across different intervals within the overall time frame. This approach is commonly used to analyze trends in diseases such as tumors, tuberculosis, HIV/AIDS, and smoking.

In this study, the Joinpoint Regression Program 5.0.2 was utilized to perform logarithmic transformations for ASIR and ASMR of TETs from 2000 to 2020. ASIR and ASMR were considered dependent variables, whereas the year (*xi*), sex, age group, race, marital status, histological subtype, and Masaoka–Koga stage were considered independent variables. A systematic observation value (*xi*, *yi*) was established, and a grid search was conducted for joinpoint analysis and parameter estimation. Model selection was performed using Monte Carlo permutation testing. The APC and average annual percentage change (AAPC) of ASIR and ASMR, along with their 95% confidence intervals (CIs), were calculated.

APC was calculated as follows: APC = (exp(*β*) − 1) × 100, where β represents the regression coefficient. AAPC was calculated as follows: AAPC = (exp(∑*wiβi*/∑*wi*) − 1) × 100, where *wi* represents the number of years included in each segment and *βi* represents the regression coefficient. APC was used to evaluate the internal trend within each independent interval, whereas AAPC was employed to assess the overall average change trend across multiple intervals. Values exceeding 0 for both APC and its 95% CI indicate an increasing trend in the incidence/mortality rate. Values lower than 0 for APC and its 95% CI suggest a decreasing trend in the incidence/mortality rate during that specific time period. A value of 0 for APC or its 95% CI indicates no significant change in the incidence/mortality rate [12]. *p* < 0.05 was considered statistically significant.

Descriptive statistical methods were used to summarize demographic and clinical characteristics. The chi‐squared test was employed to compare categorical variables. TET‐specific mortality (SM) was described as the target event, whereas mortality attributable to other causes was considered the competing event. The cumulative incidence function was employed to estimate the cumulative incidence of death, and the Nelson–Aalen cumulative risk curve was used to plot the survival curve. The Fine–Gray test was conducted for between‐group differences. Significantly different variables in univariate analysis were included in the multivariate competing risk model to determine factors related to TET‐SM. Two runs were performed, one before and one after multiple imputations. All tests were two‐sided, and *p* < 0.05 was considered statistically significant. All survival analyses were conducted using R software (version 4.3.3).

## 3 Results

### 3.1 Overall Incidence and Mortality Trends

**FIGURE 1:**
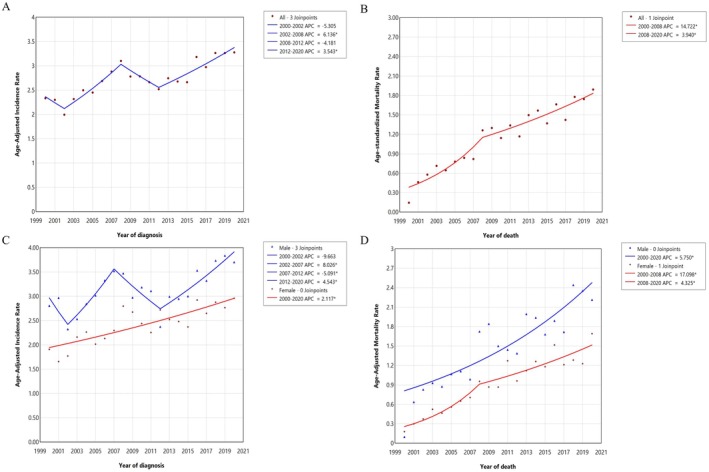
Long‐term trends in the incidence and mortality of TETs from 2000 to 2020.

From 2000 to 2020, the overall incidence and mortality of TETs were 2.769 and 1.203 per million person‐years, respectively (Table 1). During the study period, the incidence of TETs displayed a fluctuating increasing trend (Figure 1A), with a final incidence rate of 3.278 per million person‐years (Table S2). The AAPC was 1.796% (95% CI = −0.407–4.047, *p* = 0.111), but statistical significance was not reached (Table 2).

**TABLE 1:** Overall incidence and mortality from 2000 to 2020.

Variable	Incidence^a^	Mortality^a^
Overall	3.278	1.203
Sex		
Male	3.165	1.541
Female	2.444	0.955
Age at diagnosis, years		
< 60	1.509	0.383
≥ 60	9.135	5.347
Race		
White	2.337	1.024
Black	4.043	1.917
Other^b^	4.332	1.867
WHO type		
Thymoma	2.036	0.775
Thymic carcinoma	0.733	0.428
Thymoma subtype		
A	0.179	0.066
AB	0.352	0.093
B1	0.245	0.081
B2	0.309	0.087
B3	0.330	0.137
Masaoka–Koga stage		
I/IIA	0.807	0.217
IIB	1.190	0.512
III/IV	0.630	0.402

^a^ Rates were calculated as the number of cases per 1,000,000 person‐years and adjusted for age by Segi's world standard population.

^b^ Includes American Indians/Alaskan Natives and Asian/Pacific Islanders.

**TABLE 2:** Trends in the incidence and mortality of TETs by subgroup from 2000 to 2020.

Variable	Incidence			Mortality		
	AAPC (%)	95% CI	*p*	AAPC (%)	95% CI	*p*
Overall	1.796	−0.407–4.047	0.111	8.125	5.266–11.062	< 0.001
Sex						
Male	1.394	−1.034–3.882	0.263	5.750	4.022–7.508	< 0.001
Female	2.117	1.424–2.814	< 0.001	9.258	6.099–12.511	< 0.001
Age at diagnosis, years						
< 60	0.956	0.270–1.648	0.009	3.424	1.441–5.446	0.002
≥ 60	2.371	1.491–3.259	< 0.001	9.522	6.333–12.807	< 0.001
Race						
White	1.165	0.543–1.790	0.001	7.543	4.338–10.847	< 0.001
Black	2.607	1.187–4.047	0.001	6.547	4.410–8.727	< 0.001
Other^a^	1.574	0.214–2.953	0.025	7.147	4.900–9.443	< 0.001
WHO type						
Thymoma	0.308	−1.811–2.472	0.778	5.593	4.037–7.171	< 0.001
Thymic carcinoma	4.752	3.585–5.932	< 0.001	17.324	−0.423–38.235	0.056
Masaoka–Koga stage						
I/IIA	5.621	−0.263–11.852	0.061	8.700	5.949–11.524	< 0.001
IIB	1.354	0.567–3.312	0.168	4.687	2.816–6.593	< 0.001
III/IV	2.905	1.619–4.208	< 0.001	6.805	5.418–8.211	< 0.001

^a^ Includes American Indians/Alaskan Natives and Asian/Pacific Islanders.

From 2002, the incidence of TETs increased significantly (APC = 6.136%, 95% CI = 2.566–9.830, *p* = 0.003) before subsequently declining, but an increasing trend was again observed from 2012. The APC was 3.543% (95% CI = 2.090–5.018, *p* < 0.001, Table S3). The mortality of TETs continued to increase during the study period (Figure 1B), with a final incidence of 1.892 per million person‐years (Table S4), and the AAPC was 8.125% (95% CI = 4.022–7.508, *p* < 0.001, Table 2).

### 3.2 Trends in the Incidence and Mortality of TETs in Men and Women

The overall TETs incidence rates for men and women were 3.165 and 2.444 per million person‐years, respectively (Table 1). The incidence trend in men was generally consistent with the overall trend (Figure 1C). During the study period, the incidence rate of TETs in women remained lower than that in men (Table S2), but it displayed a continuous upward trend (AAPC = 2.117%, 95% CI = 1.424–2.814, *p* < 0.001, Figure 1C; Table 2), increasing from 0.179 per million person‐years in 2000 to 1.691 per million person‐years in 2020 (Table S2). The TET mortality rate exhibited a significant upward trend in both men and women, with the increase in mortality being larger among women (AAPC = 9.258%, 95% CI = 6.099–12.511, *p* < 0.001) than among men (AAPC = 5.750%, 95% CI = 4.022–7.508, *p* < 0.001, Figure 1D; Table 2).

### 3.3 Trends in the Incidence and Mortality of TETs in Different Age Groups

The incidence of TETs was highest in patients aged 65–69 years. Regarding sex, the incidence was highest among men aged 80–84 years and women aged 70–74 years, and the mortality rate increased with age (Figure S2). The incidence of TETs in patients aged < 60 and ≥ 60 years significantly differed, and the AAPCs in these age groups were 0.956 (95% CI = 0.280–1.648, *p* = 0.009) and 2.371 (95% CI = 1.491–3.529, *p* < 0.001), respectively (Figure S3A,B; Table 2). Differing from the stable incidence in men aged < 60 years, the incidence and mortality of TETs tended to increase in women aged < 60 years. Among patients aged < 60 years, the AAPC was 1.669 in women (95% CI = 0.723–2.624, *p* = 0.002) and 4.518 in men (95% CI = 2.061–7.034, *p* = 0.001). The incidence and mortality of TETs increased over time in both men and women aged ≥ 60 years, and the increases in incidence and mortality were larger for women than for men (Figure S3; Table S5).

### 3.4 Incidence and Mortality Trends by Ethnicity

The incidence rates for Black, White, and other ethnicities (American Indians/Alaskan Natives, Asian/Pacific Islanders) were 2.337, 4.043, and 4.332 per million person‐years, respectively (Table 1). During the study period, the incidence tended to increase in all races. However, the incidence was consistently higher in Black patients than in White patients, and the rate of increase was higher in Black patients (AAPC = 2.607%, 95% CI = 1.187–4.047, *p* = 0.001) than in White patients (AAPC = 1.574, 95% CI = 2.014–2.953, *p* = 0.025, Table 2). Although the mortality rate was higher in Black patients than in White patients during the study period, the temporal increase in mortality was larger in White patients (AAPC = 7.543%, 95% CI = 4.338–10.847, *p* < 0.001) than in Black patients (AAPC = 6.547%, 95% CI = 4.410–8.727, *p* < 0.001, Table 2). In particular, the mortality rate rapidly increased among White patients between 2000 and 2008 (APC = 14.100%, 95% CI = 5.888–22.949, *p* = 0.002, Figure S4; Table S6).

### 3.5 Incidence and Mortality Trends in Different WHO Subtypes

According to the 2021 World Health Organization classification, TETs can be divided into thymoma (types A, AB, B1, B2, and B3) and thymic carcinoma. The results illustrated that the incidence of thymoma was 2.036 per million person‐years, with the incidence being highest for type AB thymoma, whereas the incidence of thymic carcinoma was 0.733 per million person‐years (Table 1). During the study period, the incidence of thymoma exhibited a fluctuating upward trend without statistical significance (AAPC = 0.308%, 95% CI = −1.811–2.472, *p* = 0.778, Table 2). However, the incidence of thymoma again displayed an upward trend from 2011 (APC = 2.033%, 95% CI = 0.971–3.106, *p* = 0.002, Table S3). On the contrary, the incidence of thymic carcinoma increased rapidly from 0.214 per million person‐years in 2000 to 1.132 per million person‐years in 2020 (AAPC = 4.752%, 95% CI = 3.585–5.932, *p* < 0.001, Table 2). In addition, the mortality rate of thymic carcinoma increased from 0.204 per million person‐years in 2002 to 0.713 per million person‐years in 2020 (APC = 6.728%, 95% CI = 4.919–8.567, *p* < 0.001, Table S4). The increase in mortality was slightly lower for thymoma than for thymic carcinoma, and the AAPC was 5.593 (95% CI = 4.037–7.172, *p* < 0.001, Table 2; Figure S4).

### 3.6 Trends of Incidence and Mortality for Different Masaoka–Koga Stages

The incidence of stages I/IIA (AAPC = 5.621%, 95% CI = −0.263–11.852, *p* = 0.061) and IIB TETs (AAPC = 1.354%, 95% CI = −0.567–3.312, *p* = 0.168) displayed a fluctuating upward trend without statistical significance (Table 2). However, as presented in Figure S4, the incidence has increased for both stages I/IIA (APC = 4.548%, 95% CI = 1.406–7.778, *p* = 0.008) and IIB (APC = 8.113%, 95% CI = 2.881–13.612, *p* = 0.005) in recent years. Conversely, the incidence of stage III/IV TETs increased from 0.480 per million person‐years in 2000 to 0.765 per million person‐years in 2020 (AAPC = 2.905%, 95% CI = 1.619–4.208, *p* < 0.001, Table 2, Table S3). The mortality of stages I/IIA, IIB, and III/IV TETs increased during the study period. The increase in mortality was largest for stage I/IIA, rising from 0.014 per million person‐years to 0.406 per million person‐years (AAPC = 8.700%, 95% CI = 5.949–11.524, *p* < 0.001), followed by stage III/IV (AAPC = 6.805%, 95% CI = 5.418–8.211, *p* < 0.001) and stage IIB (AAPC = 4.687%, 95% CI = 2.816–6.593, *p* < 0.001, Table 2; Table S3).

### 3.7 Long‐Term Survival Outcome

In total, 4041 patients with TETs met the study inclusion criteria (Figure S1). The demographic and tumor characteristics of the study population are presented in Table 3. The median age at diagnosis was 60.0 years. Overall, the mean survival time of patients with thymus epithelial tumors was 72.4 months, with 1‐, 5‐, and 10‐year mortality rates of 9.2%, 29.8%, and 47.9%, respectively (Figure S5). Of the 4041 patients, 939 (23.2%) died of TETs. At diagnosis, ethnicity, marital status, WHO type, Masaoka‐Koga stage, tumor size, and surgical status were missing for 47 (1.2%), 182 (4.5%), 917 (22.7%), 203 (5.0%), 731 (18.1%), and 11 cases (0.3%), respectively.

**TABLE 3:** Demographic and tumor characteristics of the study population from 2000 to 2020.

Variable	Overall	2000–2006	2007–2013	2014–2020
Case	4041	1046	1321	1674
Age at diagnosis, years	60 (48, 69)	57 (45, 68)	59 (48, 68)	61 (51, 69)
Sex				
Male	2153 (53.3%)	580 (55.4%)	682 (51.6%)	891 (53.2%)
Female	1888 (46.7%)	466 (44.6%)	639 (48.4%)	783 (46.8%)
Age group at diagnosis				
< 60	1994 (49.3%)	587 (56.1%)	666 (50.4%)	741 (44.3%)
≥ 60	2047 (50.7%)	459 (43.9%)	655 (49.6%)	933 (55.7%)
Race				
White	2642 (65.4%)	742 (70.9%)	860 (65.1%)	1040 (62.1%)
Black	604 (14.9%)	140 (13.4%)	206 (15.6%)	258 (15.4%)
Other	748 (18.5%)	161 (15.4%)	242 (18.3%)	345 (20.6%)
Unknown	47 (1.2%)	3 (0.3%)	13 (1.0%)	31 (1.9%)
Marital status				
Single	755 (18.7%)	187 (17.9%)	254 (19.2%)	314 (18.8%)
Married	2390 (59.1%)	634 (60.6%)	742 (56.2%)	1014 (60.6%)
Separated/divorced	396 (9.8%)	97 (9.3%)	138 (10.4%)	161 (9.6%)
Widowed	318 (7.9%)	96 (9.2%)	114 (8.6%)	108 (6.5%)
Unknown	182 (4.5%)	32 (3.1%)	73 (5.5%)	77 (4.6%)
WHO type				
A/AB	776 (19.2%)	160 (15.3%)	248 (18.8%)	368 (22.0%)
B1	367 (9.1%)	97 (9.3%)	119 (9.0%)	151 (9.0%)
B2	459 (11.4%)	71 (6.8%)	145 (11.0%)	243 (14.5%)
B3	484 (12.0%)	133 (12.7%)	176 (13.3%)	175 (10.5%)
TC	1038 (25.7%)	209 (20.0%)	332 (25.1%)	497 (29.7%)
Unknown	917 (22.7%)	376 (35.9%)	301 (22.8%)	240 (14.3%)
Masaoka–Koga stage				
I/IIA	1149 (28.4%)	221 (21.1%)	384 (29.1%)	544 (32.5%)
IIB	1759 (43.5%)	550 (52.6%)	556 (42.1%)	653 (39.0%)
III/IV	930 (23.0%)	208 (19.9%)	311 (23.5%)	411 (24.6%)
Unknown	203 (5.0%)	67 (6.4%)	70 (5.3%)	66 (3.9%)
Tumor size				
≤ 5 cm	1019 (25.2%)	228 (21.8%)	302 (22.9%)	489 (29.2%)
5–10 cm	1773 (43.9%)	402 (38.4%)	629 (47.6%)	742 (44.3%)
> 10 cm	518 (12.8%)	126 (12.0%)	153 (11.6%)	239 (14.3%)
Unknown	731 (18.1%)	290 (27.7%)	237 (17.9%)	204 (12.2%)
Surgery				
No	993 (24.6%)	234 (22.4%)	340 (25.7%)	419 (25.0%)
Yes	3037 (75.2%)	807 (77.2%)	978 (74.0%)	1252 (74.8%)
Unknown	11 (0.3%)	5 (0.5%)	3 (0.2%)	3 (0.2%)
Chemotherapy				
No/unknown	2584 (63.9%)	684 (65.4%)	838 (63.4%)	1062 (63.4%)
Yes	1457 (36.1%)	362 (34.6%)	483 (36.6%)	612 (36.6%)
Radiotherapy				
No/unknown	2110 (52.2%)	460 (44.0%)	699 (52.9%)	951 (56.8%)
Yes	1931 (47.8%)	586 (56.0%)	622 (47.1%)	723 (43.2%)

In the single‐factor analysis of the competing risk model, the TET‐SM was significantly higher in older patients than in younger patients (*p* = 0.015), whereas the TET‐SM was lower in married patients than in unmarried patients (*p* = 0.024). Additionally, patients with B1, B2, or B3 thymoma and those with thymic carcinoma had higher TET‐SM than those with type A/AB thymoma (*p* < 0.001). Furthermore, the TET‐SM increased with increasing Masaoka–Koga stage (*p* < 0.001). The TET‐SM significantly increased with an increase in tumor maximum diameter (*p* < 0.001). Patients who underwent surgical treatment had a significantly lower TET‐SM than those who did not undergo surgery (*p* < 0.001, Figure S6).

Using data before multiple imputation for the competing risk model analysis, the hazard ratios (HRs) for B1, B2, and B3 thymoma and thymic carcinoma were 2.19 (95% CI = 1.43–3.35, *p* < 0.001), 1.65 (95% CI = 1.05–2.61, *p* = 0.030), 1.80 (95% CI = 1.19–2.74, *p* = 0.006), and 4.91 (95% CI = 3.40–7.10, *p* < 0.001), respectively. The TET‐SM was significantly higher in patients with advanced‐stage tumors than in those with low‐stage tumors, and the HR increased with increasing stage, particularly for stages IIB and III (Table 4).

**TABLE 4:** Multifactor competitive risk model analysis of patients with TETs before and after multiple imputation.

Variable	Before multiple imputation				After multiple imputation			
	HR	95% CI		*p*	HR	95% CI		*p*
		Upper	Lower			Upper	Lower	
Age at diagnosis								
< 60	Reference				Reference			
≥ 60	1.18	0.97	1.44	0.100	1.18	1.03	1.36	0.020
Marital status								
Single	Reference				Reference			
Married	0.80	0.63	1.03	0.082	0.85	0.72	1.01	0.068
Separated/divorced	0.95	0.67	1.35	0.770	0.96	0.75	1.24	0.770
Widowed	1.28	0.86	1.91	0.220	1.20	0.91	1.59	0.200
WHO type								
A/AB	Reference				Reference			
B1	2.19	1.43	3.35	< 0.001	1.91	1.46	2.51	< 0.001
B2	1.65	1.05	2.61	0.030	1.69	1.29	2.22	< 0.001
B3	1.80	1.19	2.74	0.006	1.90	1.47	2.44	< 0.001
TC	4.91	3.42	7.14	< 0.001	3.98	3.15	5.02	< 0.001
Masaoka–Koga stage								
I/IIA	Reference				Reference			
IIB	2.26	1.60	3.20	< 0.001	2.03	1.59	2.57	< 0.001
III/IV	3.84	2.62	5.63	< 0.001	3.26	2.52	4.21	< 0.001
Tumor size								
≤ 5 cm	Reference				Reference			
5–10 cm	1.34	1.04	1.73	0.024	1.35	1.11	1.63	0.002
> 10 cm	2.31	1.70	3.15	< 0.001	1.97	1.57	2.48	< 0.001
Surgery								
No	Reference				Reference			
Yes	0.47	0.38	0.60	< 0.001	0.38	0.33	0.44	< 0.001
Chemotherapy								
No/unknown	Reference				Reference			
Yes	1.49	1.17	1.91	0.001	1.50	1.27	1.77	< 0.001

## 4 Discussion

The incidence of TETs has apparently shifted in recent years. However, because of the rarity of these tumors, their changes in incidence and mortality over time are unclear. Thus, we conducted the largest population‐based epidemiological study of TETs to date, revealing recent trends in its incidence and mortality.

TETs are extremely rare in children and young adults, and their incidence increases in middle age, peaking at 65–69 years old. This pattern mirrors the increased age‐related incidence of many other cancers and potentially reflects the accumulation of genetic damage with age [7]. Nevertheless, the increase in the incidence of TETs with aging is in stark contrast to the progressive degeneration of the thymus with age [23]. Our results illustrated that the incidence of TETs was higher in men than in women. However, the incidence increased significantly in women during the study period, and the peak age was lower in women than in men, suggesting a clear sex difference in the incidence of TETs. A previous study in the US reported a higher incidence of TETs in males [4]. In the future, we need to explore important reasons for the significant differences in the incidence of TETs between men and women.

Our analysis revealed that the incidence of thymus cancer gradually increased from 2000 to 2020. We hypothesize that this trend might be partially attributable to advances in immunohistochemical techniques and modifications in the classification of thymus cancers. The diagnosis of thymus cancer is challenging because of its heterogeneity and rarity. In recent years, immunohistochemical technology has significantly advanced, and pathological protocols have been established [24, 25, 26, 27].

For example, routine detection of immunohistochemical markers has been recommended for the diagnosis of thymoma, especially for tumors with unclear histological features. Research has found that some new immunohistochemical markers exist in most thymic cancer patients, such as the multiple markers identified by Weissferdt et al. that can be used to distinguish thymoma from thymic cancer [25, 28]. This makes it possible to accurately diagnose the cases that were difficult to diagnose, thus leading to an increase in the number of thymic cancer diagnoses, thereby improving the overall incidence rate of TETs.

Meanwhile, the development of imaging technology has also had a significant impact on the diagnosis of TETs. The widespread application of computed tomography (CT) in clinical practice, especially in lung cancer screening, may increase the chance of detecting TETs. Although the data from this study do not directly support this viewpoint, other studies have shown that the detection rate of TETs has increased in populations undergoing low‐dose spiral CT lung cancer screening. This means that more sensitive imaging methods may be one of the important factors in the increase of the incidence rate of TETs.

Population structure change is also an important factor affecting the incidence rate and mortality of TETs. Currently, population aging has become a global trend. TETs are more common in the elderly population, and as the average life expectancy of the population increases, the proportion of elderly people gradually increases, which expands the high‐risk population for TETs. In this study, we observed that the incidence rate and mortality of TETs in patients aged ≥ 60 years were significantly higher than those in patients aged < 60 years. Therefore, population aging has promoted the incidence rate and mortality of TETs in the general population to a certain extent.

In addition, environmental factors and lifestyle changes may also have an impact on the incidence rate and mortality of TETs, although there is no conclusive study to prove their direct correlation. Carcinogens in the environment, such as certain chemicals and radiation, may increase the risk of TETs. Changes in lifestyle, such as smoking, drinking, lack of exercise, long‐term mental stress, etc., may affect the function of the immune system, thereby affecting the occurrence and development of tumors. Taking smoking as an example, smoking is an important risk factor for various cancers, which may increase the risk of TETs by affecting the body's immune surveillance and cell repair mechanisms. However, further research is needed to confirm the specific roles of these factors in TETs. The effect of age on the survival of patients with TETs remains controversial. Ruffini et al. reported that increasing age is a predictor of shortened survival in thymus cancer [29]. A retrospective analysis of 797 patients with TETs identified age as a risk factor affecting prognosis [30]. However, Rea et al. [31] found that only WHO histology and Masaoka–Koga stage were independent prognostic factors in a multi‐factor analysis including sex, age, adjuvant radiotherapy, surgery, Masaoka–Koga stage, and WHO histological classification. Gripp et al. [32] revealed that age did not influence survival outcomes among patients with TETs. The retrospective designs and limited sample sizes of prior studies might have contributed to the controversy. The main factor contributing to these differing results was the age range in previously published papers. Therefore, in our study, we included patients of all ages and found that age is an important prognostic factor for TETs.

Although OS is the most commonly reported measure of probability of survival, cancer‐specific mortality might be more meaningful for TETs because it excludes non‐TETs causes of death and provides more disease‐specific prognostic factors [33]. We retrospectively analyzed all patients with confirmed TETs in the SEER database over the period of 2000–2020. Through competitive risk model analysis, we found that the Masaoka–Koga stage, the WHO tissue type, tumor size, and surgery were correlated with survival. Surgical resection is considered the primary treatment for TETs. The role of adjuvant radiotherapy or adjuvant chemoradiotherapy after surgery is controversial, especially for stage II disease. Although the use of adjuvant radiotherapy has been reported to improve OS, most previous studies combined stages II and III. In our study, patients with adjuvant chemotherapy had poorer prognoses, possibly because all patients with stage IV disease and most patients with stage III disease received adjuvant therapy.

The study results suggest that the WHO histological type is an important prognostic factor for TETs. This is consistent with the results of Guerrera et al. [34], emphasizing the importance of the WHO tissue type in TETs. In clinical practice, the WHO classification can be used to divide patients with TETs into low‐ (types A, AB, and B1) and high‐risk recurrence groups (type B2, type B3, and thymic carcinoma). Tseng et al. [35] reported that the Masaoka–Koga stage, the WHO tissue type, tumor size, the receipt of adjuvant therapy, and the incisal margin status were important factors affecting the recurrence of TETs, and the recurrence rates were higher for types B2 and B3 than for types A, AB, and B1. Okumura et al. [36] also believed that type B2 and B3 tumors were more malignant than type A, AB, and B1 tumors. However, in our study, the prognosis of type B1 thymoma was worse than that of types A and AB. Many studies have confirmed that there is a close relationship between thymoma and MG, and the correlation between type B thymoma and MG is more prominent. As an important paraneoplastic syndrome of TETs, MG may have adverse effects on tumor prognosis by affecting the immune function and body status of patients. Although there are currently no studies that directly compare the prognostic differences between B1 thymomas with or without MG and A and AB thymomas, this prediction is reasonable based on existing studies [37, 38].

Our results highlighted tumor size as an important prognostic factor for TETs, and TET‐SM increased with increasing tumor size. From the perspective of clinical practice, tumor size has a direct impact on surgical outcomes. Almost all reports on the surgical treatment of TETs identified total resection as an independent prognostic factor. R0 resection might be more difficult for larger tumors, which is also an important factor leading to poor prognosis [17]. In addition, larger tumors tend to have more extensive blood loss and a greater need for transfusion during surgery, and immune regulation associated with transfusion could lead to worse tumor outcomes [39]. From the perspective of oncology, relatively large tumors tend to have greater tumor aggressiveness and higher tumor proliferation indices, and they might have more unfavorable tumor biological behavior [40]. From the perspective of biological behavior, larger tumor size could be linked to more aggressive tumor behavior. Prior studies identified a positive correlation between tumor necrosis and tumor size, and tumor necrosis often portends a poor prognosis [40]. Further analysis revealed an association between tumor size and Masaoka‐Koga classification. In Masaoka‐Koga stage, the proportion of small tumors (≤ 5 cm) in stage I/IIA was relatively high, the proportion of medium‐sized tumors (5–10 cm) in stage IIB was increased, and the proportion of large tumors (> 10 cm) in stage III/IV was significantly increased. This suggests that the increase in tumor size with stage progression may be associated with a more advanced disease state and poorer prognosis.

The study had several limitations. First, this was a retrospective study, resulting in inevitable selection bias. In addition, the degree of surgical removal, mode of surgery, and presence of MG were not reported in the SEER database. The exact Masaoka–Koga staging information is not listed in the SEER database, and this staging was inferred from several existing variables in our research. Finally, The absence of approximately 20% of WHO classification and tumor size data is an important limitation of this study. Although we used multiple filling methods and the data analysis results were basically consistent before and after interpolation, these data are key factors in the prognosis of TETs, and the absence of these data may lead to inaccurate analysis of prognostic factors, affecting the accurate judgment of the relationship between WHO classification and TET‐SM and the relationship between tumor size and prognosis. Therefore, further research is needed to confirm our findings.

## 5 Conclusion

The present study revealed that the incidence and mortality of TETs have increased significantly in recent years, and this trend is mainly attributable to the rapid growth in its incidence and mortality in women. In addition, the peak age was approximately 10 years younger in women than in men. The incidence of TETs was higher in Asian/Pacific Islander and Black patients than in White patients. Both thymoma and thymic carcinoma displayed an increasing mortality rate over time. This study not only revealed trends in TETs incidence and mortality, but also identified multiple prognostic factors independently associated with TET‐specific mortality, including age, WHO histological type, Masaoka‐Koga stage, tumor size, surgery, and chemotherapy. This suggests that early diagnosis, combined with effective treatment such as surgery, is critical to improving the prognosis of patients with TETs.

## Author Contributions

**Zishan Chen:** conceptualization (lead), data curation (lead), formal analysis (lead), methodology (lead), visualization (lead), writing – original draft (lead), project administration (lead). **Shiwen Liu:** methodology (supporting), visualization (supporting), writing – review and editing (supporting). **Chunting Chen:** methodology (supporting), writing – review and editing (supporting). **Jinmang Zhuang, Xinying Xu**, and **Maolin Liu:** investigation (equal), writing – review and editing (equal). **Fancai Lai** and **Fei He:** conceptualization, methodology, writing – review and editing, supervision (supporting).

## Ethics Statement

The data were taken from a public database, and thus, ethical approval was not required.

## Consent

The authors have nothing to report.

## Conflicts of Interest

The authors declare no conflicts of interest.

## Supporting information


Data S1.

## Data Availability

The data that support the findings of this study are openly available in the SEER database.

## References

[1] M. Li , F. Hou , J. Zhao , et al., “Focal Adhesion Kinase Is Overexpressed in Thymic Epithelial Tumor and May Serve as an Independent Prognostic Biomarker,” Oncology Letters 15, no. 3 (2018): 3001–3007. 29435030 10.3892/ol.2017.7676 PMC5778861

[2] E. A. Engels , “Epidemiology of Thymoma and Associated Malignancies,” Journal of Thoracic Oncology 5, no. 10 (2010): S260–S265. 20859116 10.1097/JTO.0b013e3181f1f62d PMC2951303

[3] W. K. de Jong , J. L. Blaauwgeers , M. Schaapveld , W. Timens , T. J. Klinkenberg , and H. J. Groen , “Thymic Epithelial Tumours: A Population‐Based Study of the Incidence, Diagnostic Procedures and Therapy,” European Journal of Cancer 44, no. 1 (2008): 123–130. 18068351 10.1016/j.ejca.2007.11.004

[4] C. H. Hsu , J. K. Chan , C. H. Yin , C. C. Lee , C. U. Chern , and C. I. Liao , “Trends in the Incidence of Thymoma, Thymic Carcinoma, and Thymic Neuroendocrine Tumor in the United States,” PLoS One 14, no. 12 (2019): e0227197. 31891634 10.1371/journal.pone.0227197 PMC6938371

[5] A. Marx , J. K. C. Chan , L. Chalabreysse , et al., “The 2021 WHO Classification of Tumors of the Thymus and Mediastinum: What Is New in Thymic Epithelial, Germ Cell, and Mesenchymal Tumors?,” Journal of Thoracic Oncology 17, no. 2 (2022): 200–213. 34695605 10.1016/j.jtho.2021.10.010

[6] M. Scorsetti , F. Leo , A. Trama , et al., “Thymoma and Thymic Carcinomas,” Critical Reviews in Oncology/Hematology 99 (2016): 332–350. 26818050 10.1016/j.critrevonc.2016.01.012

[7] K. Kondo and Y. Monden , “Therapy for Thymic Epithelial Tumor: A Clinical Study of 1,320 Patients From Japan,” Annals of Thoracic Surgery 76, no. 3 (2003): 878–884. Discussion 884–5. 12963221 10.1016/s0003-4975(03)00555-1

[8] N. Girard , C. Du Merveilleux Vignaux , T. Molina , B. Besse , and R. Rythmic , “Thymic Tumors,” Revue de Pratique 67, no. 4 (2017): 430–434. 30512890

[9] M. Chiappetta , F. Lococo , L. Pogliani , et al., “Masaoka‐Koga and TNM Staging System in Thymic Epithelial Tumor: Prognostic Comparison and the Role of the Number of Involved Structures,” Cancers (Basel) 13, no. 21 (2021): 5254. 34771417 10.3390/cancers13215254 PMC8582470

[10] K. Koga , Y. Matsuno , M. Noguchi , et al., “A Review of 79 Thymomas: Modification of Staging System and Reappraisal of Conventional Division Into Invasive and Non‐Invasive Thymoma,” Pathology International 44, no. 5 (1994): 359–367. 8044305 10.1111/j.1440-1827.1994.tb02936.x

[11] J. K. Yun , H. R. Kim , D. K. Kim , Y. M. Shim , Y. T. Kim , and K. Y. Chung , “Tumor Size as a Prognostic Factor in Limited‐Stage Thymic Epithelial Tumor: A Multicenter Analysis,” Journal of Thoracic and Cardiovascular Surgery 162, no. 1 (2021): 309–317.e9. 32736865 10.1016/j.jtcvs.2020.05.048

[12] J. O. Lee , G. D. Lee , H. R. Kim , et al., “An Overview of Surgical Treatment of Thymic Epithelial Tumor in Korea: A Retrospective Multicenter Analysis,” Journal of Thoracic Surgery 55, no. 2 (2022): 126–142. 10.5090/jcs.21.124 PMC9005939 35370141

[13] M. Knetki‐Wróblewska , D. M. Kowalski , M. Olszyna‐Serementa , M. Krzakowski , and M. Szołkowska , “Thymic Epithelial Tumor: Do We Know All the Prognostic Factors?,” Thoracic Cancer 12, no. 3 (2021): 339–348. 33386778 10.1111/1759-7714.13750 PMC7862797

[14] T. Fukui , Y. Kadomatsu , H. Tsubouchi , et al., “Prognostic Factors of Stage I Thymic Epithelial Tumor,” General Thoracic and Cardiovascular Surgery 69, no. 1 (2021): 59–66. 32621280 10.1007/s11748-020-01427-x

[15] T. Fukui , K. Fukumoto , T. Okasaka , et al., “Prognostic Impact of Tumour Size in Completely Resected Thymic Epithelial Tumours,” European Journal of Cardio‐Thoracic Surgery 50, no. 6 (2016): 1068–1074. 27999073 10.1093/ejcts/ezw178

[16] M. Okumura , I. Yoshino , M. Yano , et al., “Tumour Size Determines Both Recurrence‐Free Survival and Disease‐Specific Survival After Surgical Treatment for Thymoma,” European Journal of Cardio‐Thoracic Surgery 56, no. 1 (2019): 174–181. 30783650 10.1093/ejcts/ezz001

[17] Q. Liu , X. Fu , X. Su , et al., “Elevated Pretreatment Serum Lactate Dehydrogenase Level Predicts Inferior Overall Survival and Disease‐Free Survival After Resection of Thymic Carcinoma,” Journal of Thoracic Disease 9, no. 11 (2017): 4550–4560. 29268525 10.21037/jtd.2017.10.86 PMC5720965

[18] M. W. Kang , E. S. Lee , J. Jo , et al., “Stage III Thymic Epithelial Neoplasms Are Not Homogeneous With Regard to Clinical, Pathological, and Prognostic Features,” Journal of Thoracic Oncology 4, no. 12 (2009): 1561–1567. 19745769 10.1097/JTO.0b013e3181b9cd7f

[19] R. J. Rieker , J. Hoegel , A. Morresi‐Hauf , et al., “Histologic Classification of Thymic Epithelial Tumors: Comparison of Established Classification Schemes,” International Journal of Cancer 98, no. 6 (2002): 900–906. 11948470 10.1002/ijc.10255

[20] S. Siesling , J. M. van der Zwan , I. Izarzugaza , et al., “Rare Thoracic Cancers, Including Peritoneum Mesothelioma,” European Journal of Cancer 48, no. 7 (2012): 949–960. 22406029 10.1016/j.ejca.2012.02.047

[21] A. T. Fernandes , E. T. Shinohara , M. Guo , et al., “The Role of Radiation Therapy in Malignant Thymoma: A Surveillance, Epidemiology, and End Results Database Analysis,” Journal of Thoracic Oncology 5, no. 9 (2010): 1454–1460. 20651611 10.1097/JTO.0b013e3181e8f345

[22] P. C. Austin , I. R. White , D. S. Lee , and S. van Buuren , “Missing Data in Clinical Research: A Tutorial on Multiple Imputation,” Canadian Journal of Cardiology 37, no. 9 (2021): 1322–1331. 33276049 10.1016/j.cjca.2020.11.010 PMC8499698

[23] A. A. Gal , M. J. Kornstein , C. Cohen , I. G. Duarte , J. I. Miller , and K. A. Mansour , “Neuroendocrine Tumors of the Thymus: A Clinicopathological and Prognostic Study,” Annals of Thoracic Surgery 72, no. 4 (2001): 1179–1182. 11603433 10.1016/s0003-4975(01)03032-6

[24] P. Gaur , C. Leary , and J. C. Yao , “Thymic Neuroendocrine Tumors: A SEER Database Analysis of 160 Patients,” Annals of Surgery 251, no. 6 (2010): 1117–1121. 20485130 10.1097/SLA.0b013e3181dd4ec4

[25] F. C. Detterbeck , C. Moran , J. Huang , et al., “Which Way Is Up? Policies and Procedures for Surgeons and Pathologists Regarding Resection Specimens of Thymic Malignancy,” Zhongguo Fei Ai Za Zhi 17, no. 2 (2014): 95–103. 24581159 10.3779/j.issn.1009-3419.2014.02.06 PMC6131236

[26] A. Marchevsky , A. Marx , P. Strobel , et al., “Policies and Reporting Guidelines for Small Biopsy Specimens of Mediastinal Masses,” Zhongguo Fei Ai Za Zhi 17, no. 2 (2014): 104–109. 24581160 10.3779/j.issn.1009-3419.2014.02.07 PMC6131233

[27] A. Marx , R. Rieker , A. Toker , F. Länger , and P. Ströbel , “Thymic Carcinoma: Is It a Separate Entity? From Molecular to Clinical Evidence,” Thoracic Surgery Clinics 21, no. 1 (2011): 25–31. v‐vi. 21070984 10.1016/j.thorsurg.2010.08.010

[28] A. Weissferdt and C. A. Moran , “Immunohistochemistry in the Diagnosis of Thymic Epithelial Neoplasms,” Applied Immunohistochemistry & Molecular Morphology 22, no. 7 (2014): 479–487. 24897066 10.1097/PAI.0b013e3182a53856

[29] E. Ruffini , F. Detterbeck , D. Van Raemdonck , et al., “Tumours of the Thymus: A Cohort Study of Prognostic Factors From the European Society of Thoracic Surgeons Database,” European Journal of Cardio‐Thoracic Surgery 46, no. 3 (2014): 361–368. 24482389 10.1093/ejcts/ezt649 PMC4155438

[30] P. L. Filosso , A. Evangelista , E. Ruffini , et al., “Does Myasthenia Gravis Influence Overall Survival and Cumulative Incidence of Recurrence in Thymoma Patients? A Retrospective Clinicopathological Multicentre Analysis on 797 Patients,” Lung Cancer 88, no. 3 (2015): 338–343. 25819383 10.1016/j.lungcan.2015.03.007

[31] F. Rea , G. Marulli , R. Girardi , et al., “Long‐Term Survival and Prognostic Factors in Thymic Epithelial Tumours,” European Journal of Cardio‐Thoracic Surgery 26, no. 2 (2004): 412–418. 15296907 10.1016/j.ejcts.2004.04.041

[32] S. Gripp , K. Hilgers , R. Wurm , and G. Schmitt , “Thymoma: Prognostic Factors and Treatment Outcomes,” Cancer 83, no. 8 (1998): 1495–1503. 9781943

[33] E. Kim and C. R. Thomas, Jr. , “Conditional Survival of Malignant Thymoma Using National Population‐Based Surveillance, Epidemiology, and End Results (SEER) Registry (1973–2011),” Journal of Thoracic Oncology 10, no. 4 (2015): 701–707. 25590603 10.1097/JTO.0000000000000472

[34] F. Guerrera , E. A. Rendina , F. Venuta , et al., “Does the World Health Organization Histological Classification Predict Outcomes After Thymomectomy? Results of a Multicentre Study on 750 Patients,” European Journal of Cardio‐Thoracic Surgery 48, no. 1 (2015): 48–54. 25246487 10.1093/ejcts/ezu368

[35] Y. C. Tseng , Y. H. Tseng , H. L. Kao , et al., “Correction: Long Term Oncological Outcome of Thymoma and Thymic Carcinoma – An Analysis of 235 Cases From a Single Institution,” PLoS One 12, no. 9 (2017): e0185399. 28931090 10.1371/journal.pone.0185399 PMC5607199

[36] M. Okumura , M. Ohta , H. Tateyama , et al., “The World Health Organization Histologic Classification System Reflects the Oncologic Behavior of Thymoma: A Clinical Study of 273 Patients,” Cancer 94, no. 3 (2002): 624–632. 11857293 10.1002/cncr.10226

[37] İ. Altınönder , M. Kaya , S. P. Yentür , et al., “Thymic Gene Expression Analysis Reveals a Potential Link Between HIF‐1A and Th17/Treg Imbalance in Thymoma Associated Myasthenia Gravis,” Journal of Neuroinflammation 21, no. 1 (2024): 126. 38734662 10.1186/s12974-024-03095-7 PMC11088784

[38] T. G. Blum , D. Misch , J. Kollmeier , S. Thiel , and T. T. Bauer , “Autoimmune Disorders and Paraneoplastic Syndromes in Thymoma,” Journal of Thoracic Disease 12, no. 12 (2020): 7571–7590. 33447448 10.21037/jtd-2019-thym-10 PMC7797875

[39] N. Dusch , C. Weiss , P. Ströbel , P. Kienle , S. Post , and M. Niedergethmann , “Factors Predicting Long‐Term Survival Following Pancreatic Resection for Ductal Adenocarcinoma of the Pancreas: 40 Years of Experience,” Journal of Gastrointestinal Surgery 18, no. 4 (2014): 674–681. 24241965 10.1007/s11605-013-2408-x

[40] C. C. Ko , C. H. Chang , T. Y. Chen , et al., “Solid Tumor Size for Prediction of Recurrence in Large and Giant Non‐Functioning Pituitary Adenomas,” Neurosurgical Review 45, no. 2 (2022): 1401–1411. 34606021 10.1007/s10143-021-01662-7 PMC8976796

