# Supplementary material for: Rising Threat: Long‐Term Trends in the Incidence and Mortality of Thymic Epithelial Tumor
Source: Cancer Med. 2025 May 15;14(10):e70968. doi: 10.1002/cam4.70968 (PMC12079087; doi:10.1002/cam4.70968)
Supplement: Supplementary file 1 — Data S1. [file CAM4-14-e70968-s001.docx]

Supplementary Figure 1. Standard flow chart for inclusion and exclusion of patients with thymic epithelial tumors in the SEER database, 2000-2020

All patients with thymic epithelial tumors were identified using site codes (C37.9) and ICD-O-3 oncology codes (8580/3-8585/3, 8020/3, 8023/3, 8033/3, 8070/3, 8082/3, 8123/3, 8140/3, 8144/3, 8200/3, 8260/3, 8310/3, 8430/3, 8560/3, 8586/3, 8980/3) and cases were histologically or cytologically confirmed. Only the first match was selected for each individual. These cases are included in our analysis of incidence and mortality trends (n=4979)

Excluded:

Non-first or non-sole malignant tumor, diagnosis sequence unknown (N=847)

Cases diagnosed based on autopsy or death certificate (N=4)

Cases with missing cause of death (N=35)

Cases diagnosed less than 1 month before death (N=52);

Included in our descriptive and survival analysis (n=4041)

Supplementary table 1. Comparison between Masaoka-Koga staging system and SEER staging system

| Masaoka–Koga Staging System | Stage Groupings from SEER Data (Adapted from Fernandes et al.) |
| --- | --- |
| I: Grossly and microscopically completely encapsulated tumor | “Localized”: “invasive carcinoma confined to gland of origin” or “localized, not otherwise specified” |
| IIA: Microscopic transcapsular invasion |  |
| IIB: Macroscopic invasion into thymic or surrounding fatty tissue, or grossly adherent to but not breaking through mediastinal pleura or pericardium | “Regional”: “adjacent connective tissue” |
|  |  |
| III: Macroscopic invasion into neighboring organ (i.e., pericardium, great vessel, or lung) | Distant”: “adjacent organs/structures” or “further contiguous extension” or any positive lymph nodes |
| IVA: Pleural or pericardial metastases |  |
| IVB: Lymphogenous or hematogenous metastasis |  |

Supplementary table 2. Age-standardized incidence of thymus epithelial tumors in different subgroups from 2000 to 2020

| Year | Overall | Sex | | Age at diagnosis, y | | Race | | | WHO organization type | | Masaoka-Koga stage | | |
| --- | --- | --- | --- | --- | --- | --- | --- | --- | --- | --- | --- | --- | --- |
|  |  | Male | Female | <60 | ≥60 | White | Black | Other | Thymoma | Thymic carcinoma | I&IIA | IIB | III&IV |
| 2000 | 2.334 | 2.806 | 0.179 | 1.433 | 6.892 | 2.095 | 3.122 | 3.512 | 2.120 | 0.214 | 0.430 | 1.226 | 0.480 |
| 2001 | 2.301 | 2.969 | 0.298 | 1.477 | 6.470 | 1.943 | 3.573 | 3.855 | 1.844 | 0.457 | 0.477 | 1.112 | 0.580 |
| 2002 | 1.995 | 2.324 | 0.374 | 1.175 | 6.140 | 1.768 | 2.838 | 2.885 | 1.500 | 0.495 | 0.355 | 1.075 | 0.371 |
| 2003 | 2.318 | 2.535 | 0.524 | 1.373 | 7.093 | 2.066 | 2.813 | 3.889 | 1.905 | 0.412 | 0.532 | 1.177 | 0.475 |
| 2004 | 2.499 | 2.844 | 0.465 | 1.433 | 7.886 | 2.365 | 3.174 | 2.596 | 1.842 | 0.656 | 0.641 | 1.251 | 0.441 |
| 2005 | 2.452 | 3.018 | 0.559 | 1.398 | 7.775 | 2.223 | 2.638 | 3.795 | 1.942 | 0.510 | 0.536 | 1.312 | 0.485 |
| 2006 | 2.688 | 3.328 | 0.652 | 1.491 | 8.742 | 2.473 | 3.262 | 3.643 | 2.113 | 0.576 | 0.684 | 1.485 | 0.440 |
| 2007 | 2.882 | 3.520 | 0.706 | 1.600 | 9.363 | 2.334 | 3.924 | 5.682 | 2.288 | 0.594 | 0.834 | 1.322 | 0.560 |
| 2008 | 3.102 | 3.473 | 0.955 | 1.503 | 11.183 | 2.442 | 5.423 | 5.352 | 2.332 | 0.770 | 1.033 | 1.215 | 0.663 |
| 2009 | 2.782 | 2.975 | 0.868 | 1.611 | 8.698 | 2.393 | 4.691 | 3.496 | 2.067 | 0.714 | 0.853 | 1.231 | 0.568 |
| 2010 | 2.785 | 3.185 | 0.867 | 1.679 | 8.375 | 2.256 | 3.911 | 4.880 | 2.099 | 0.686 | 0.690 | 1.020 | 0.915 |
| 2011 | 2.664 | 3.111 | 1.273 | 1.318 | 9.464 | 2.431 | 3.604 | 3.363 | 1.882 | 0.782 | 0.723 | 1.233 | 0.599 |
| 2012 | 2.521 | 2.375 | 0.963 | 1.235 | 9.020 | 2.129 | 3.748 | 3.639 | 1.885 | 0.636 | 0.788 | 1.120 | 0.530 |
| 2013 | 2.746 | 2.995 | 1.120 | 1.585 | 8.610 | 2.255 | 3.683 | 4.935 | 1.955 | 0.791 | 0.824 | 0.964 | 0.693 |
| 2014 | 2.678 | 2.950 | 1.262 | 1.500 | 8.635 | 2.293 | 3.217 | 4.789 | 1.929 | 0.749 | 0.850 | 1.053 | 0.675 |
| 2015 | 2.664 | 3.002 | 1.181 | 1.446 | 8.819 | 2.334 | 3.034 | 4.427 | 1.983 | 0.681 | 0.906 | 0.960 | 0.695 |
| 2016 | 3.183 | 3.531 | 1.517 | 1.515 | 11.614 | 2.806 | 4.736 | 4.051 | 2.189 | 0.994 | 1.180 | 1.083 | 0.662 |
| 2017 | 2.975 | 3.323 | 1.213 | 1.740 | 9.215 | 2.169 | 5.546 | 5.470 | 2.087 | 0.887 | 1.110 | 1.056 | 0.640 |
| 2018 | 3.265 | 3.735 | 1.284 | 1.663 | 11.359 | 2.697 | 5.373 | 4.261 | 2.195 | 1.069 | 0.914 | 1.364 | 0.904 |
| 2019 | 3.264 | 3.838 | 1.228 | 1.830 | 10.511 | 2.582 | 4.974 | 5.193 | 2.273 | 0.990 | 0.986 | 1.388 | 0.820 |
| 2020 | 3.278 | 3.704 | 1.691 | 1.580 | 11.856 | 2.637 | 5.002 | 4.680 | 2.145 | 1.132 | 1.090 | 1.348 | 0.765 |

Supplementary table 3. Annual percentage change in incidence of thymus epithelial tumors, 2000-2020

| Variable | Time period | APC（%） | 95%CI | *P* |
| --- | --- | --- | --- | --- |
|  |  |  |  |  |
| Overall | 2000-2002 | -5.305 | -5.305--19.836 | 0.483 |
|  | 2002-2008 | 6.136 | 2.566-9.830 | 0.003 |
|  | 2008-2012 | -4.181 | -10.778-2.904 | 0.212 |
|  | 2012-2020 | 3.543 | 2.090-5.018 | <0.001 |
| Sex |  |  |  |  |
| Male | 2000-2002 | -9.663 | -24.927-8.704 | 0.249 |
|  | 2002-2007 | 8.026 | 1.959-14.454 | 0.014 |
|  | 2007-2012 | -5.091 | -9.839--0.094 | 0.047 |
|  | 2012-2020 | 4.543 | 2.831-6.283 | <0.001 |
| Female | 2000-2020 | 2.117 | 1.424-2.814 | <0.001 |
| Age at diagnosis, y |  |  |  |  |
| <60 | 2000-2020 | 0.956 | 0.270-1.648 | 0.009 |
| ≥60 | 2000-2020 | 2.371 | 1.491-3.259 | <0.001 |
| Race |  |  |  |  |
| White | 2000-2020 | 1.165 | 0.543-1.790 | 0.001 |
| Black | 2000-2020 | 2.607 | 1.187-4.047 | 0.001 |
| Other^a^ | 2000-2020 | 1.574 | 0.214-2.953 | 0.025 |
| WHO organization type | |  |  |  |
| Thymoma | 2000-2002 | -11.865 | -22.690-0.475 | 0.057 |
|  | 2002-2008 | 6.262 | 3.343-9.263 | 0.001 |
|  | 2008-2011 | -7.427 | -17.912-4.399 | 0.183 |
|  | 2011-2020 | 2.033 | 0.971-3.106 | 0.002 |
| Thymic carcinoma | 2000-2020 | 4.752 | 3.585-5.932 | <0.001 |
| Thymoma subtype |  |  |  |  |
| A | 2000-2020 | 2.084 | -0.195-4.415 | 0.071 |
| AB | 2000-2020 | 4.295 | 2.319-6.308 | <0.001 |
| B1 | 2000-2020 | 0.959 | -0.882-2.835 | 0.291 |
| B2 | 2000-2020 | 6.496 | 4.190-8.854 | <0.001 |
| B3 | 2000-2020 | -0.892 | -3.189-1.459 | 0.434 |
| Masaoka-Koga stage | |  |  |  |
| I and IIA | 2000-2008 | 11.655 | 6.008-17.604 | 0.001 |
|  | 2008-2011 | -6.091 | -36.234-38.303 | 0.731 |
|  | 2011-2020 | 4.548 | 1.406-7.788 | 0.008 |
| IIB | 2000-2006 | 3.536 | -0.647-7.896 | 0.092 |
|  | 2006-2015 | -3.596 | -6.079--1.048 | 0.010 |
|  | 2015-2020 | 8.113 | 2.881-13.612 | 0.005 |
| III and IV | 2000-2020 | 2.905 | 1.619-4.208 | <0.001 |

Supplementary table 4. Age-standardized mortality rates for different subgroups of thymus epithelial tumors from 2000 to 2020

| Year | Overall | Sex | | Age at diagnosis, y | | Race | | | WHO organization type | | Masaoka-Koga stage | | |
| --- | --- | --- | --- | --- | --- | --- | --- | --- | --- | --- | --- | --- | --- |
|  |  | Male | Female | <60 | ≥60 | White | Black | Other | Thymoma | Thymic carcinoma | I&IIA | IIB | III&IV |
| 2000 | 0.145 | 0.098 | 0.179 | 0.032 | 0.718 | 0.122 | 0.463 | - | 0.029 | 0.029 | 0.014 | 0.071 | 0.045 |
| 2001 | 0.463 | 0.637 | 0.298 | 0.222 | 1.684 | 0.459 | 0.574 | 0.282 | 0.087 | 0.087 | 0.014 | 0.238 | 0.166 |
| 2002 | 0.578 | 0.829 | 0.374 | 0.294 | 2.014 | 0.473 | 1.031 | 0.939 | 0.204 | 0.204 | 0.058 | 0.261 | 0.232 |
| 2003 | 0.714 | 0.931 | 0.524 | 0.257 | 3.022 | 0.671 | 1.016 | 0.934 | 0.164 | 0.164 | 0.145 | 0.330 | 0.195 |
| 2004 | 0.646 | 0.876 | 0.465 | 0.310 | 2.343 | 0.595 | 0.842 | 0.858 | 0.276 | 0.276 | 0.053 | 0.293 | 0.272 |
| 2005 | 0.780 | 1.068 | 0.559 | 0.302 | 3.191 | 0.667 | 1.624 | 0.947 | 0.210 | 0.210 | 0.044 | 0.491 | 0.219 |
| 2006 | 0.837 | 1.112 | 0.652 | 0.295 | 3.579 | 0.791 | 0.746 | 1.082 | 0.292 | 0.292 | 0.124 | 0.458 | 0.231 |
| 2007 | 0.819 | 0.987 | 0.706 | 0.338 | 3.253 | 0.695 | 1.058 | 1.266 | 0.277 | 0.277 | 0.139 | 0.392 | 0.275 |
| 2008 | 1.262 | 1.725 | 0.955 | 0.438 | 5.428 | 1.131 | 1.628 | 1.904 | 0.393 | 0.393 | 0.256 | 0.515 | 0.397 |
| 2009 | 1.299 | 1.843 | 0.868 | 0.603 | 4.816 | 1.111 | 2.279 | 1.845 | 0.412 | 0.412 | 0.185 | 0.583 | 0.407 |
| 2010 | 1.144 | 1.502 | 0.867 | 0.367 | 5.070 | 1.021 | 1.927 | 1.451 | 0.359 | 0.359 | 0.242 | 0.470 | 0.358 |
| 2011 | 1.338 | 1.445 | 1.273 | 0.468 | 5.737 | 1.122 | 2.413 | 1.759 | 0.444 | 0.444 | 0.206 | 0.636 | 0.402 |
| 2012 | 1.169 | 1.388 | 0.963 | 0.391 | 5.098 | 0.953 | 2.147 | 1.865 | 0.436 | 0.436 | 0.142 | 0.557 | 0.386 |
| 2013 | 1.497 | 1.995 | 1.120 | 0.476 | 6.656 | 1.349 | 2.248 | 1.851 | 0.667 | 0.667 | 0.271 | 0.650 | 0.470 |
| 2014 | 1.567 | 1.939 | 1.262 | 0.441 | 7.260 | 1.235 | 2.688 | 2.810 | 0.616 | 0.616 | 0.318 | 0.604 | 0.561 |
| 2015 | 1.370 | 1.680 | 1.181 | 0.368 | 6.435 | 1.331 | 1.782 | 1.171 | 0.481 | 0.481 | 0.316 | 0.450 | 0.498 |
| 2016 | 1.663 | 1.891 | 1.517 | 0.434 | 7.877 | 1.452 | 2.479 | 2.453 | 0.611 | 0.611 | 0.264 | 0.763 | 0.536 |
| 2017 | 1.423 | 1.718 | 1.213 | 0.431 | 6.439 | 1.209 | 2.074 | 2.185 | 0.535 | 0.535 | 0.359 | 0.481 | 0.508 |
| 2018 | 1.778 | 2.443 | 1.284 | 0.384 | 8.824 | 1.548 | 2.073 | 3.139 | 0.622 | 0.622 | 0.361 | 0.718 | 0.577 |
| 2019 | 1.744 | 2.354 | 1.228 | 0.559 | 7.732 | 1.291 | 3.289 | 3.236 | 0.650 | 0.650 | 0.363 | 0.640 | 0.670 |
| 2020 | 1.892 | 2.215 | 1.691 | 0.549 | 8.677 | 1.589 | 3.035 | 2.692 | 0.713 | 0.713 | 0.406 | 0.761 | 0.628 |


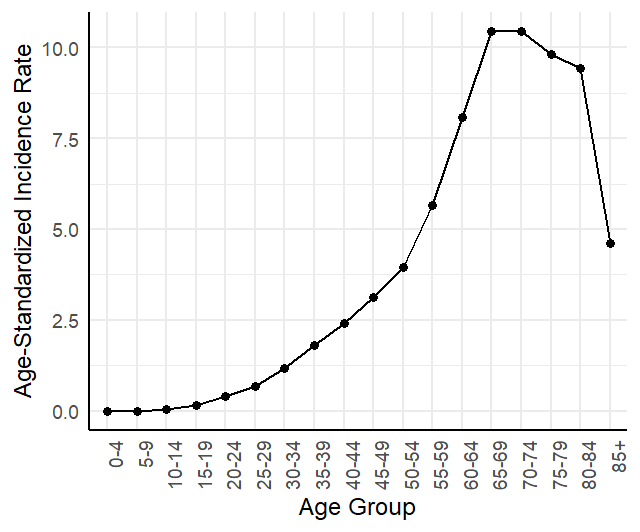

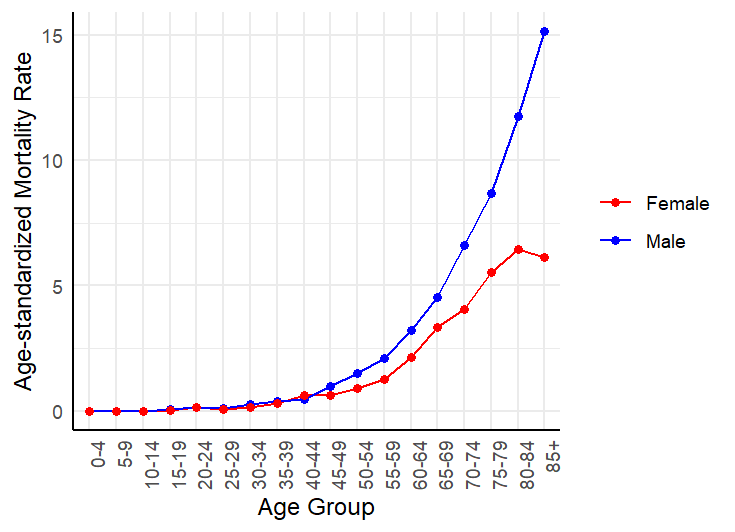


A

B

C

D


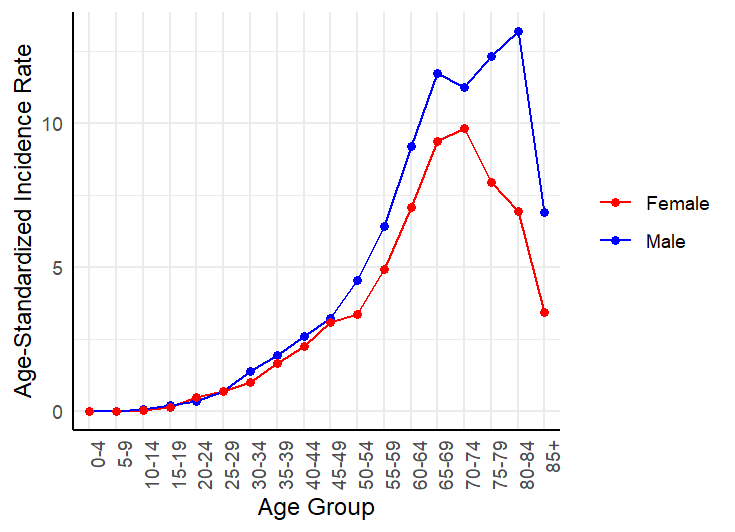

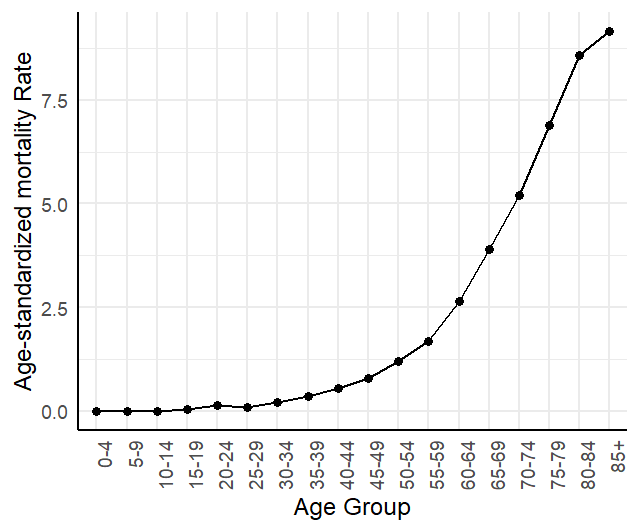


Supplementary Figure 2. Age-standardized incidence (A-B) and mortality (C-D) for different age groups, 2000-2020


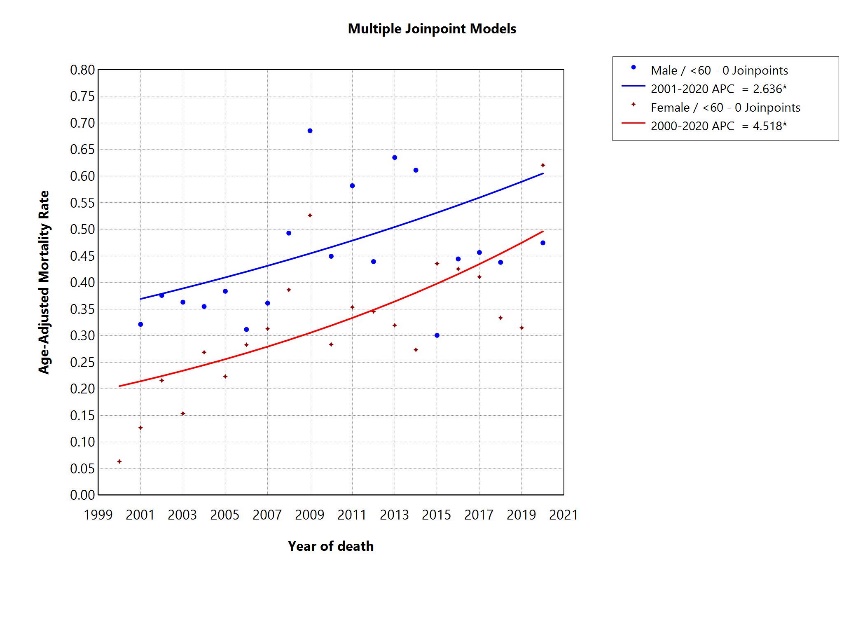


G

A

B

C

D

E

F


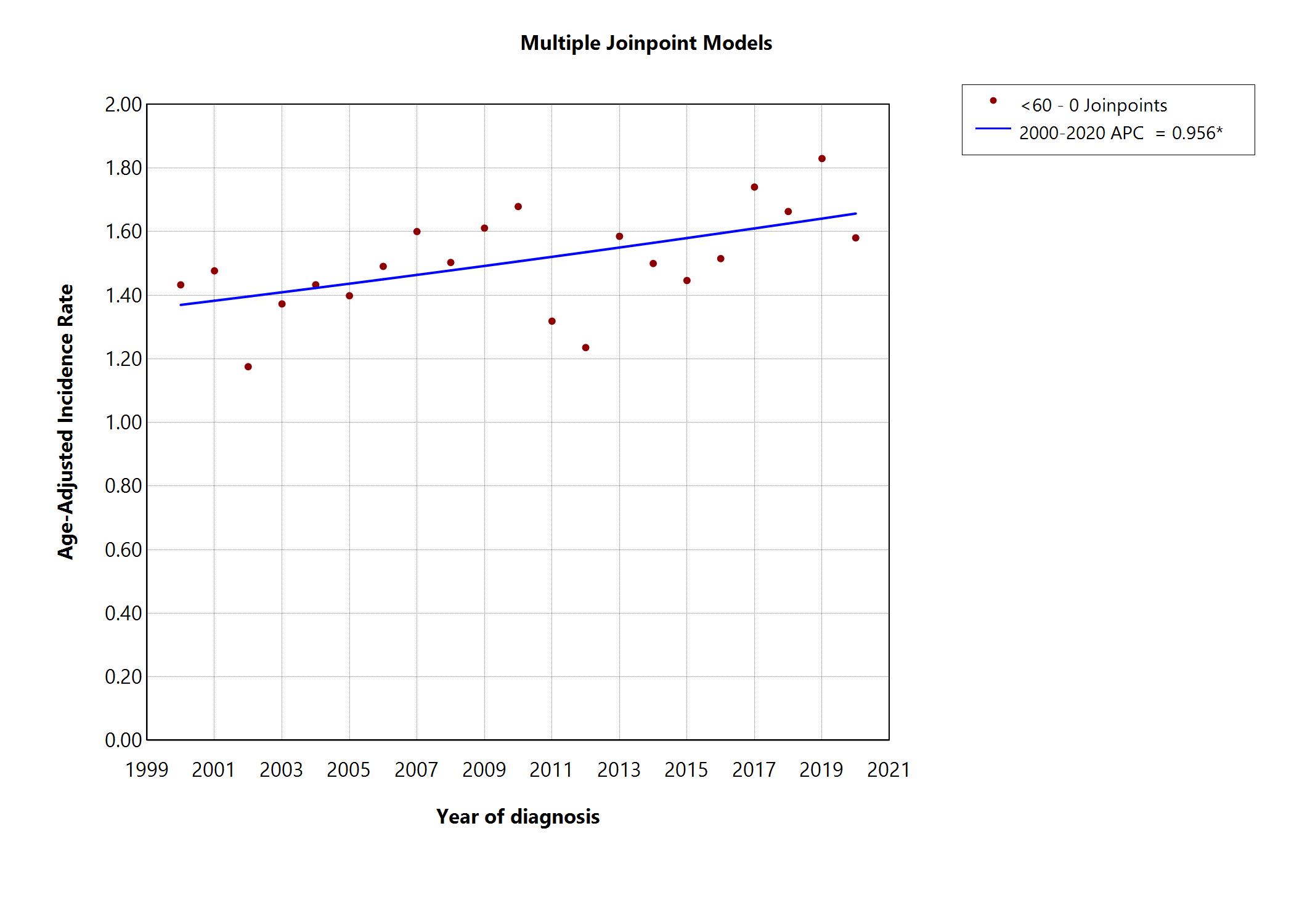

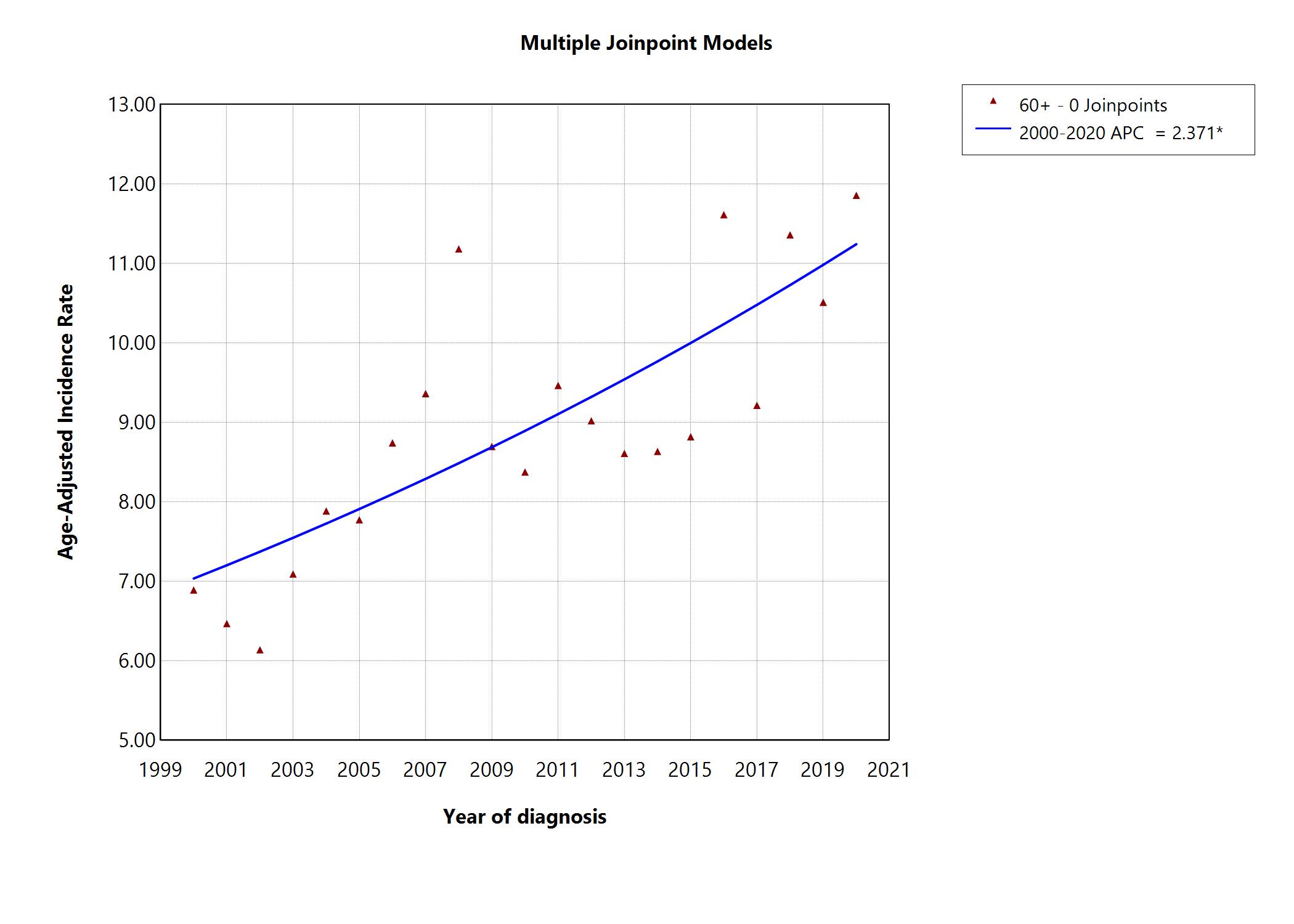

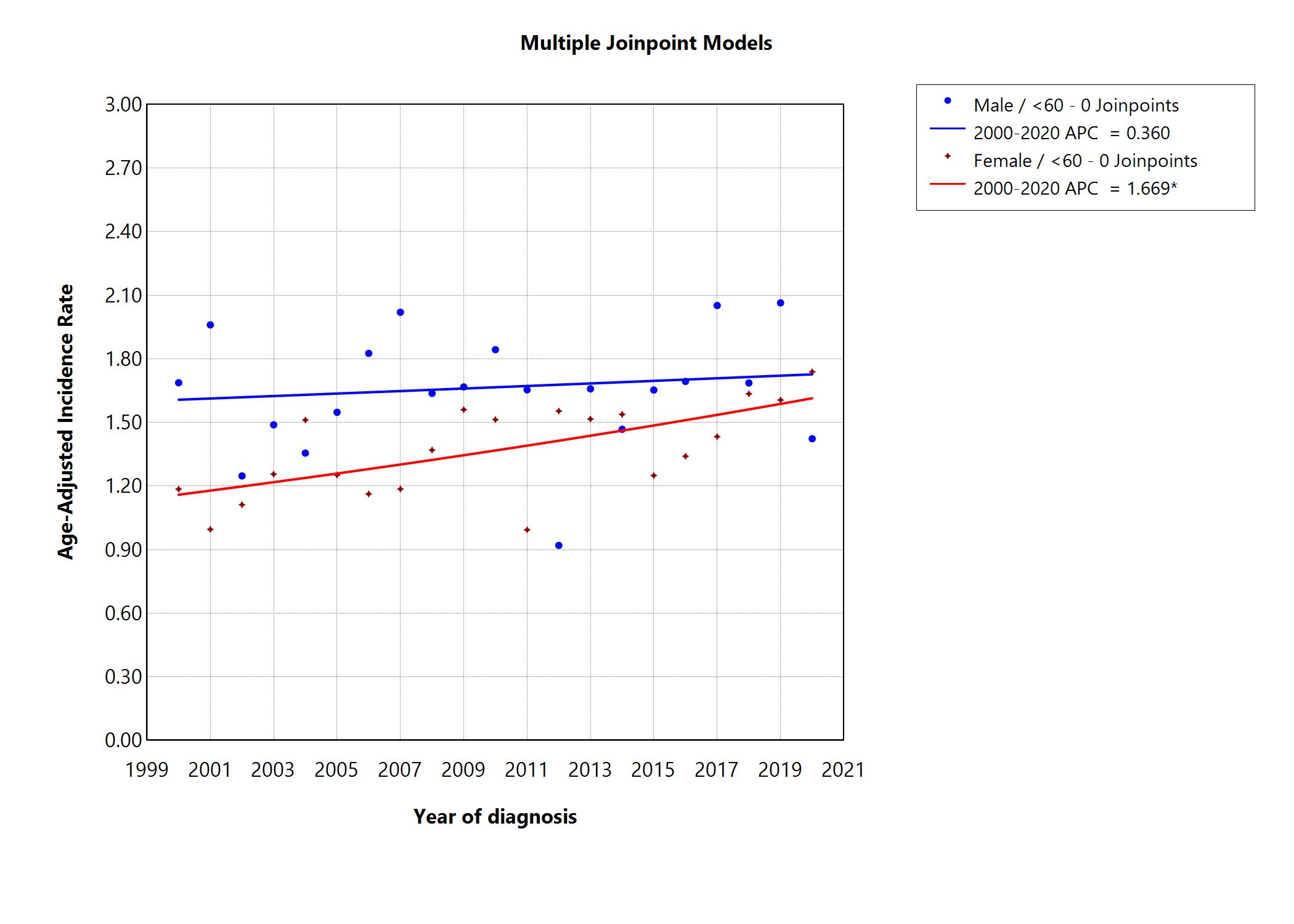

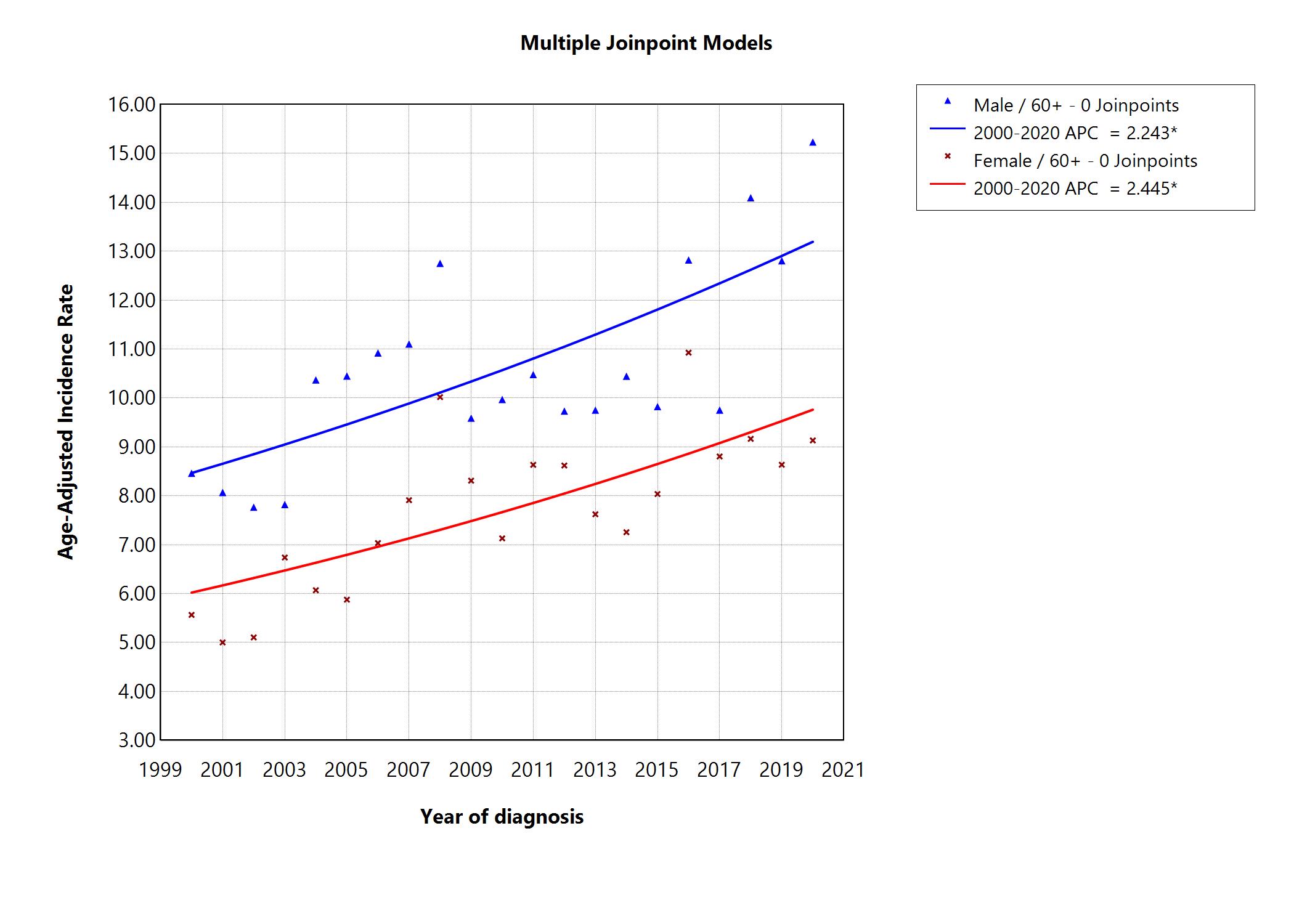

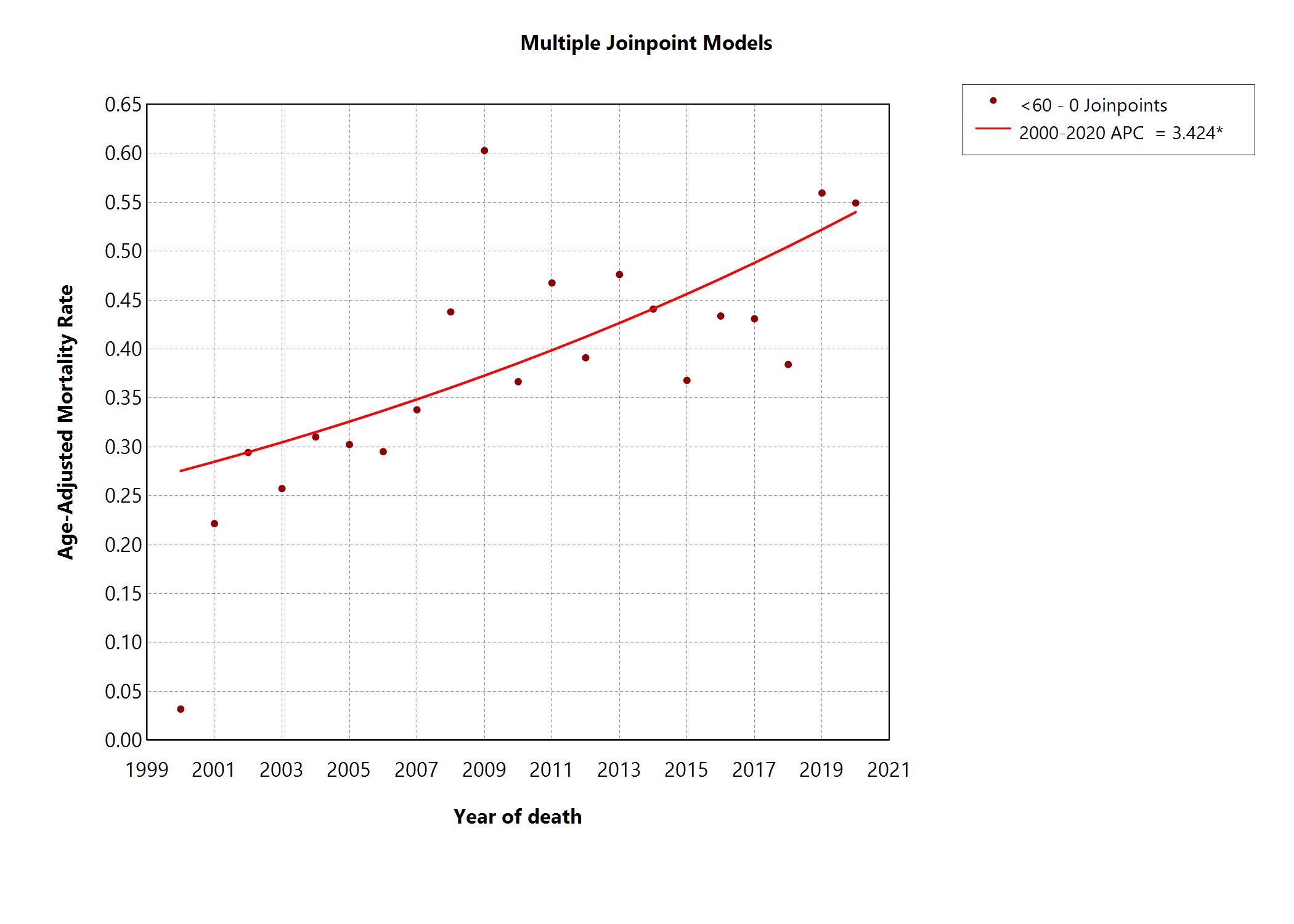

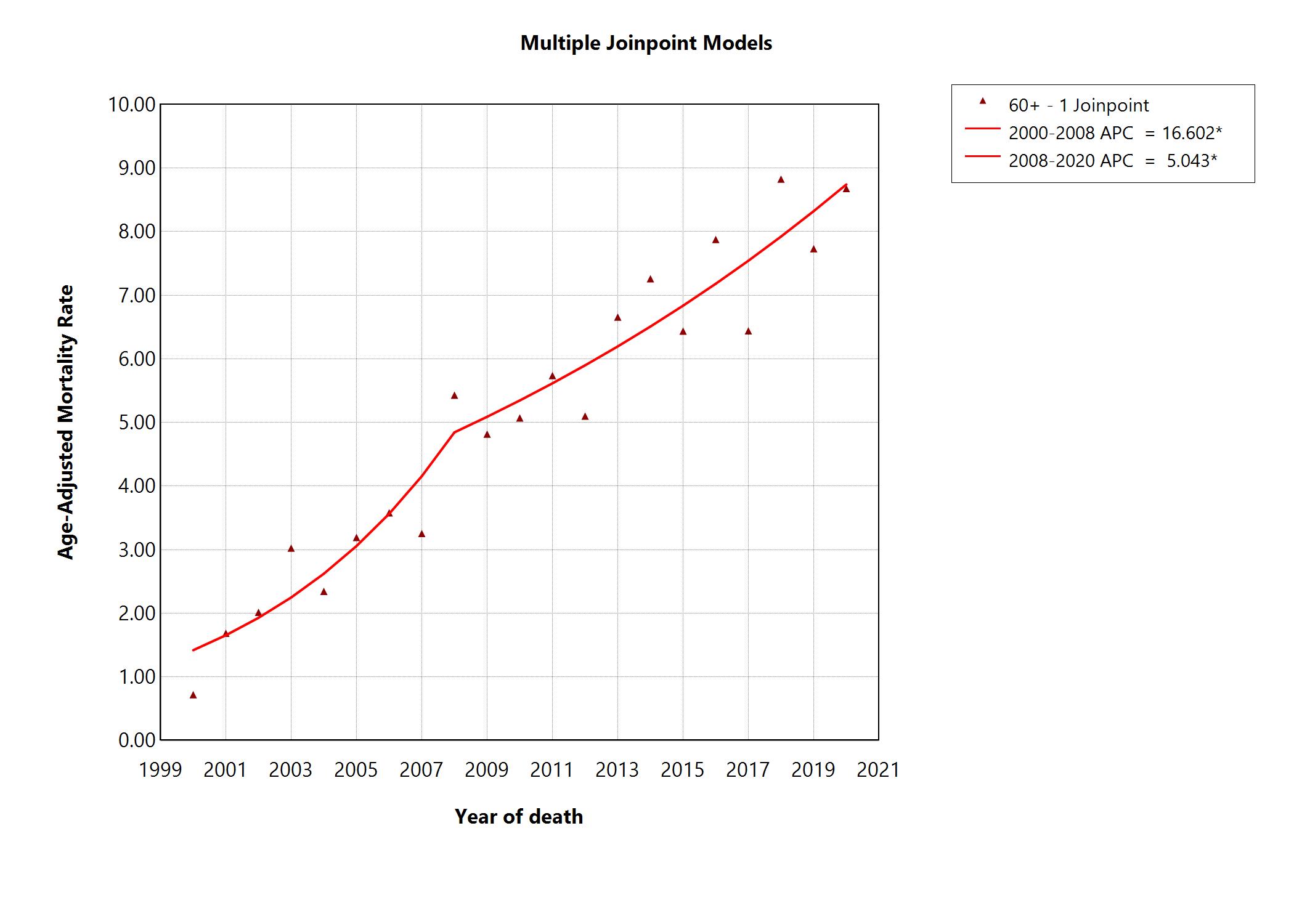


H


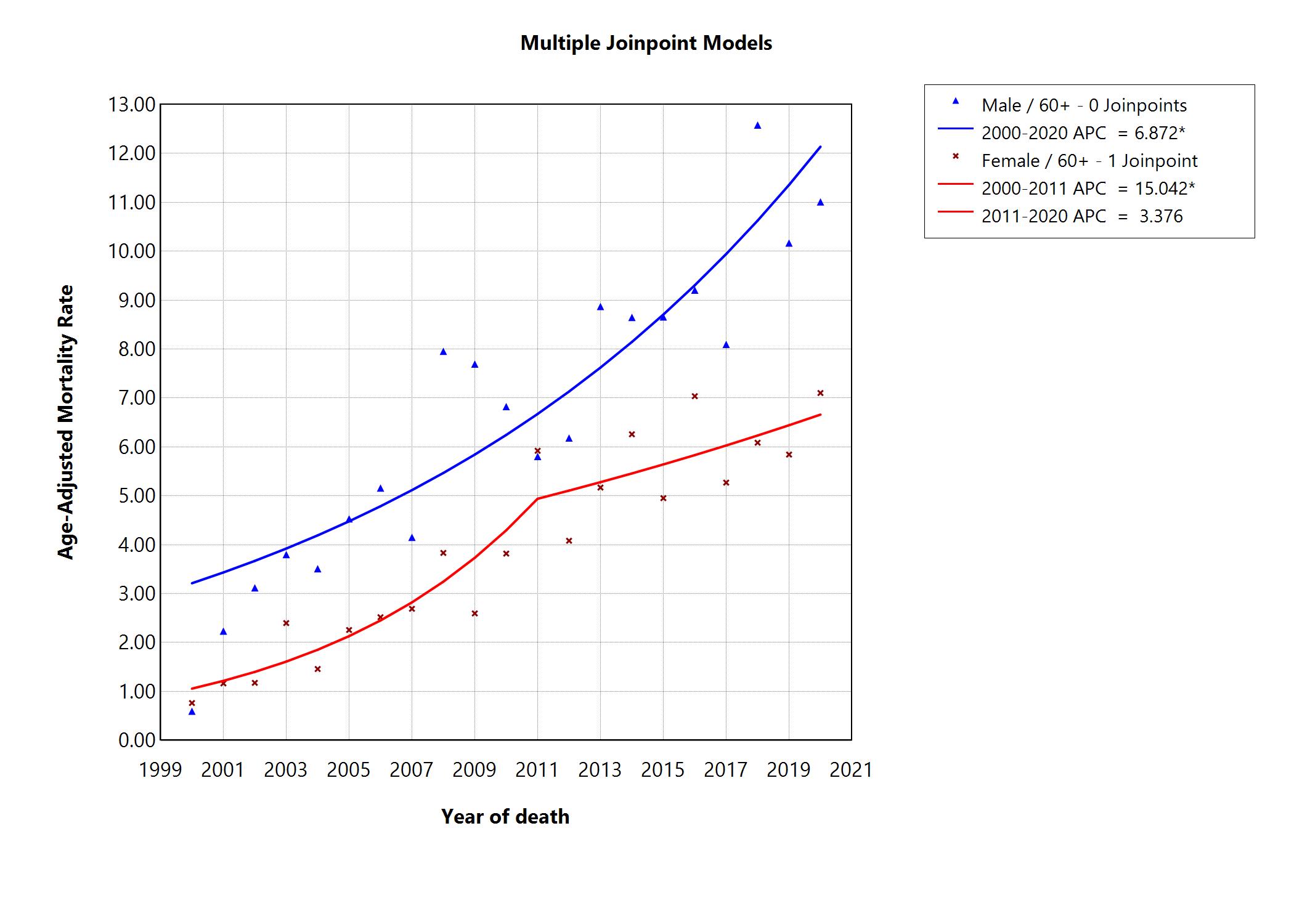


Supplementary Figure 3. Incidence(A-D) and mortality(E-H) trends for different age groups, men and women, 2000-2020

Supplementary Table 5. Incidence and mortality trends for different age groups, men and women, 2000-2020

| Variable | | Incidence | | | Mortality | | |  |
| --- | --- | --- | --- | --- | --- | --- | --- | --- |
|  |  | AAPC（%） | 95%CI | *P* | AAPC（%） | 95%CI | *P* |  |
|  |  |  |  |  |  |  |  |  |
| Male | <60 | 0.360 | -0.933-1.669 | 0.569 | 2.636 | 0.427-4.892 | 0.022 |  |
|  | ≥60 | 2.243 | 1.203-3.294 | <0.001 | 6.872 | 5.010-8.766 | <0.001 |  |
| Female | <60 | 1.669 | 0.723-2.624 | 0.002 | 4.518 | 2.061-7.034 | 0.001 |  |
|  | ≥60 | 2.445 | 1.355-3.546 | <0.001 | 9.638 | 6.305-13.075 | <0.001 |  |

A

B

C

D

E

F


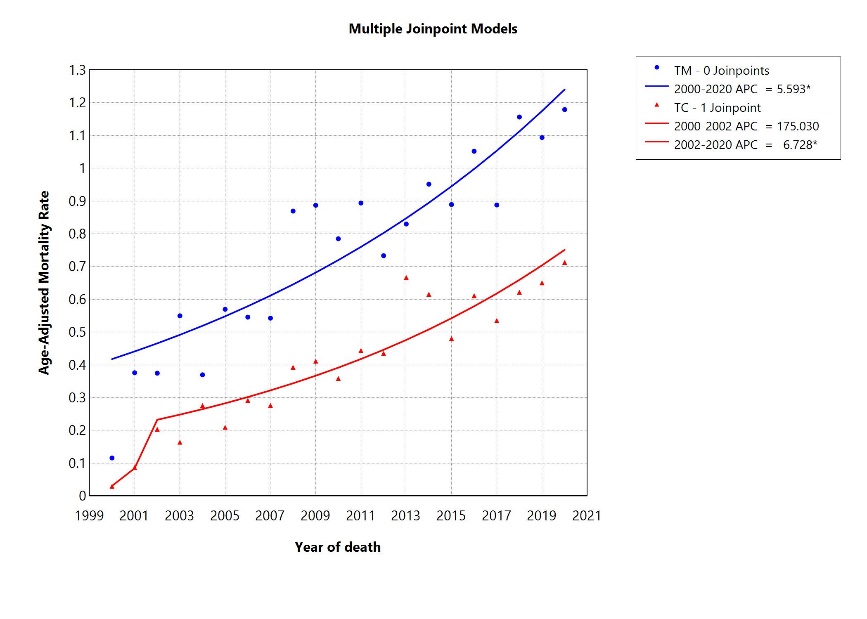

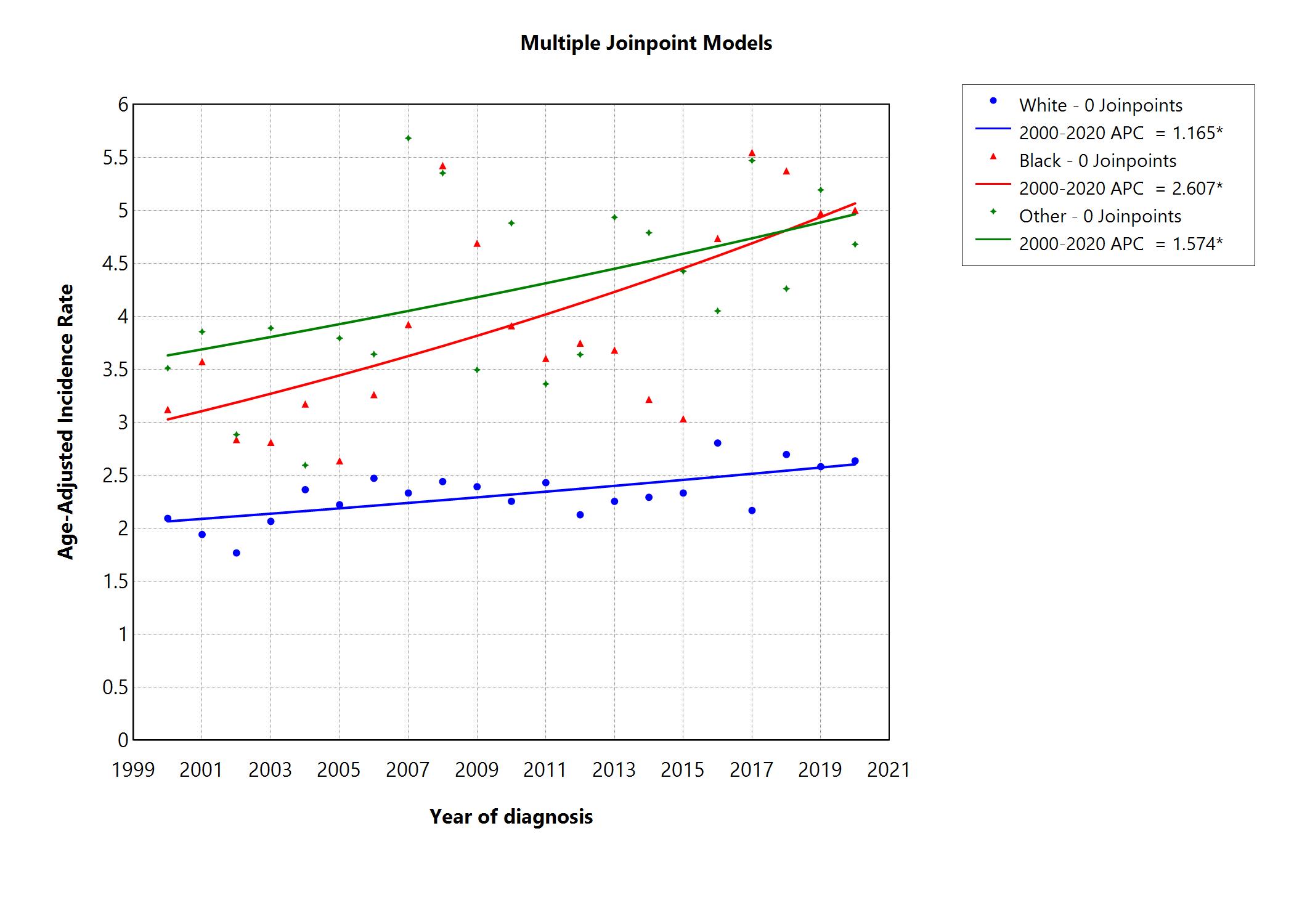

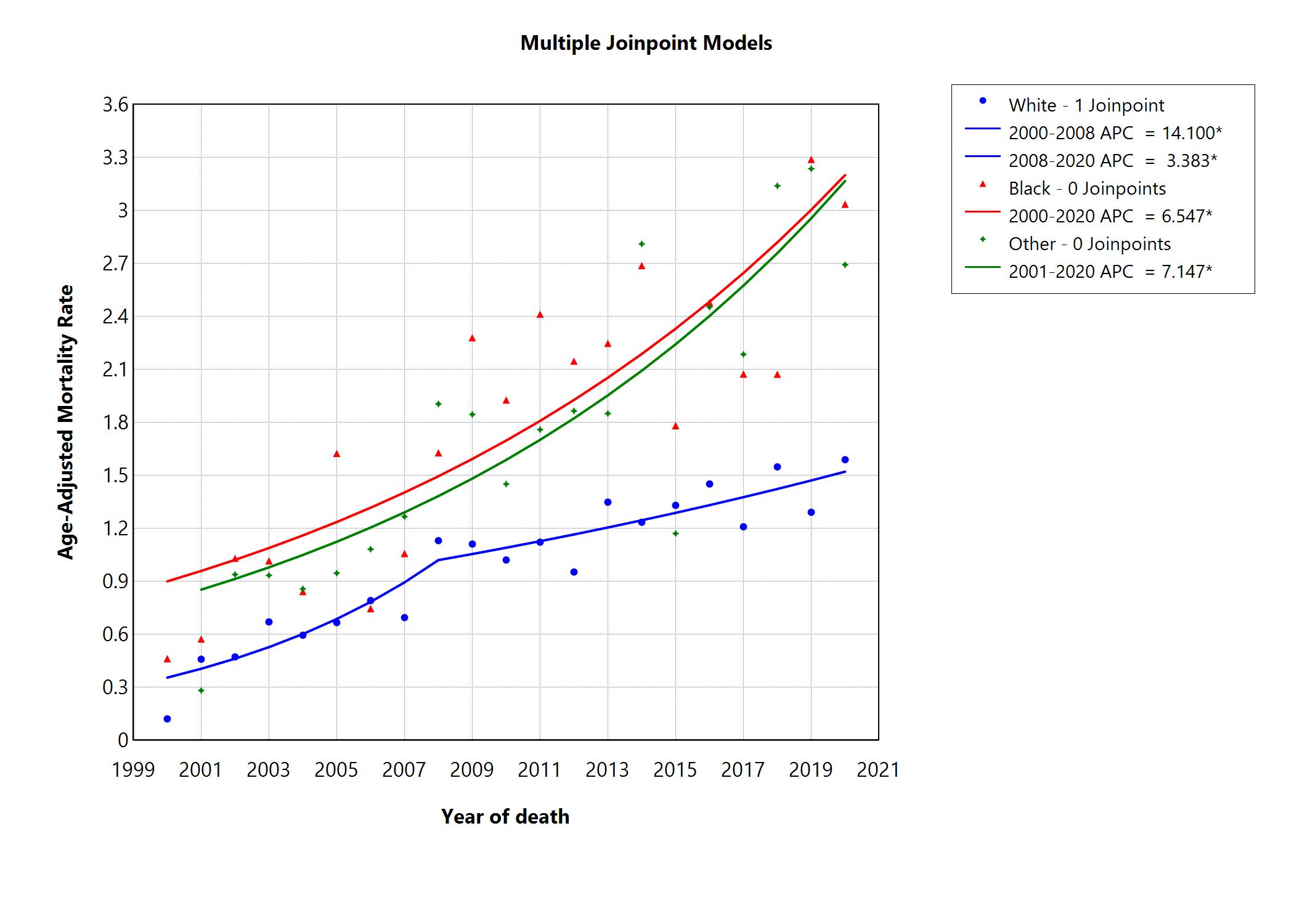

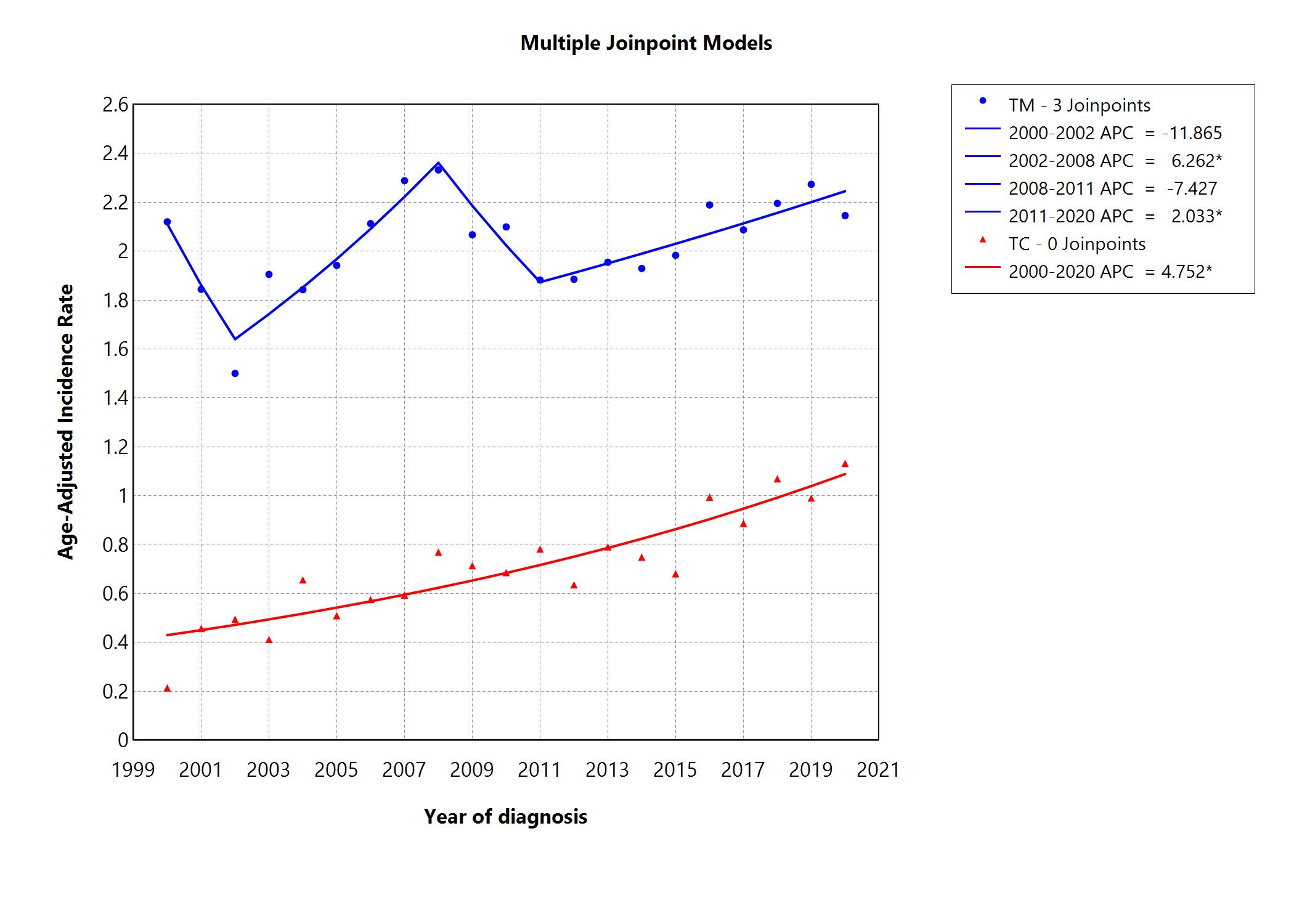

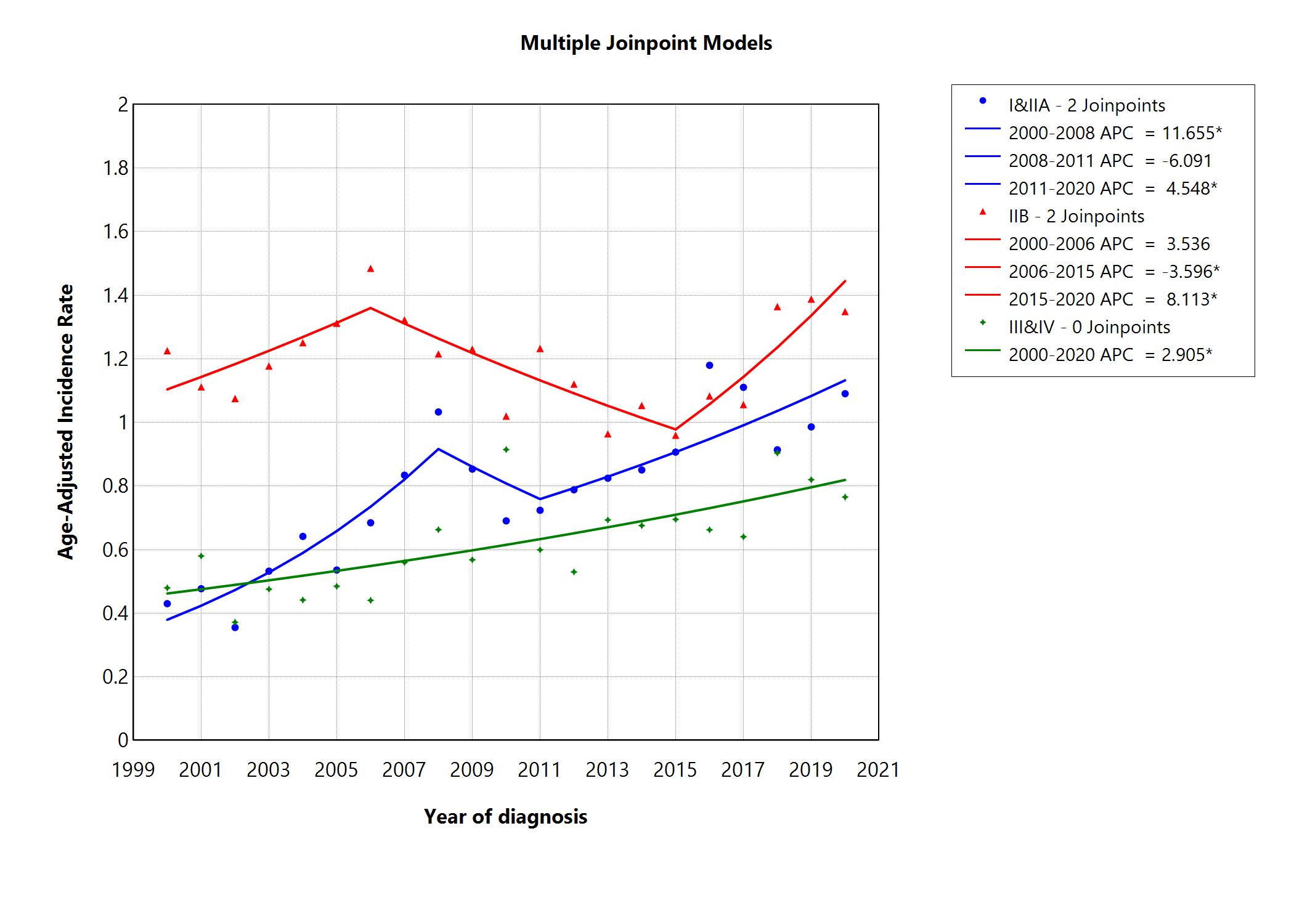

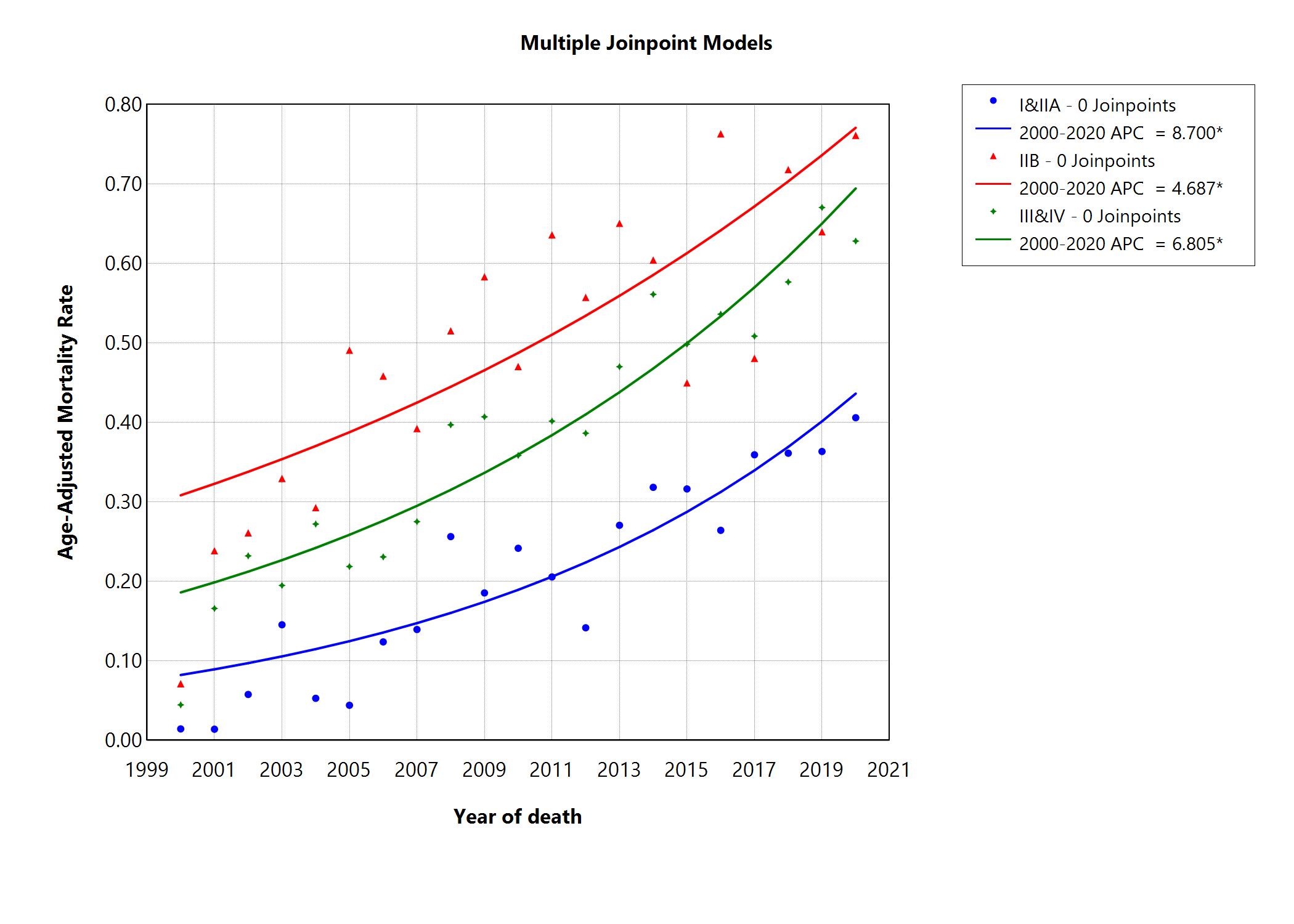


Supplementary Figure 4. Trends in incidence and mortality by ethnicity(A-B), WHO histological subtype(C-D), Masaoka-Koga staging(E-F), 2000-2020

Supplement Table 6. Annual percentage change in thymus epithelial tumor mortality from 2000 to 2020

| Variable | Time period | APC（%） | 95%CI | *P* |
| --- | --- | --- | --- | --- |
|  |  |  |  |  |
| Overall | 2000-2008 | 14.722 | 7.326-22.629 | <0.001 |
|  | 2008-2020 | 3.940 | 1.988-5.929 | 0.001 |
| Sex |  |  |  |  |
| Male | 2000-2020 | 5.750 | 4.022-7.508 | <0.001 |
| Female | 2000-2008 | 17.098 | 8.887-25.928 | <0.001 |
|  | 2008-2020 | 4.325 | 2.140-6.557 | 0.001 |
| Age at diagnosis, y |  |  |  |  |
| <60 | 2000-2020 | 3.424 | 1.441-5.446 | 0.002 |
| ≥60 | 2000-2008 | 16.602 | 8.266-25.580 | <0.001 |
|  | 2008-2020 | 5.043 | 2.983-7.144 | <0.001 |
| Race |  |  |  |  |
| White | 2000-2008 | 14.100 | 5.888-22.949 | 0.002 |
|  | 2008-2020 | 3.383 | 1.107-5.710 | 0.006 |
| Black | 2000-2020 | 6.547 | 4.410-8.727 | <0.001 |
| Other | 2000-2020 | 7.147 | 4.900-9.443 | <0.001 |
| WHO organization type |  |  |  |  |
| Thymoma | 2000-2020 | 5.593 | 4.037-7.171 | <0.001 |
| Thymic carcinoma | 2000-2002 | 175.03 | -53.026-1510.296 | 0.243 |
|  | 2002-2020 | 6.728 | 4.919-8.567 | <0.001 |
| Masaoka-Koga stage |  |  |  |  |
| I and IIA | 2000-2020 | 8.700 | 5.949-11.524 | <0.001 |
| IIB | 2000-2020 | 4.687 | 2.816-6.593 | <0.001 |
| III and IV | 2000-2020 | 6.805 | 5.418-8.211 | <0.001 |


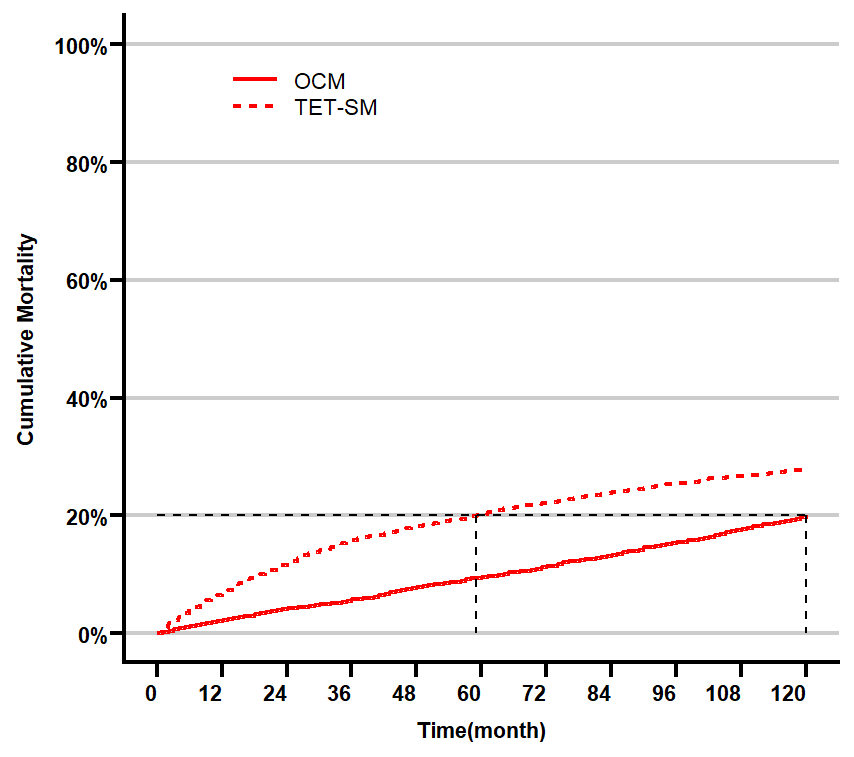

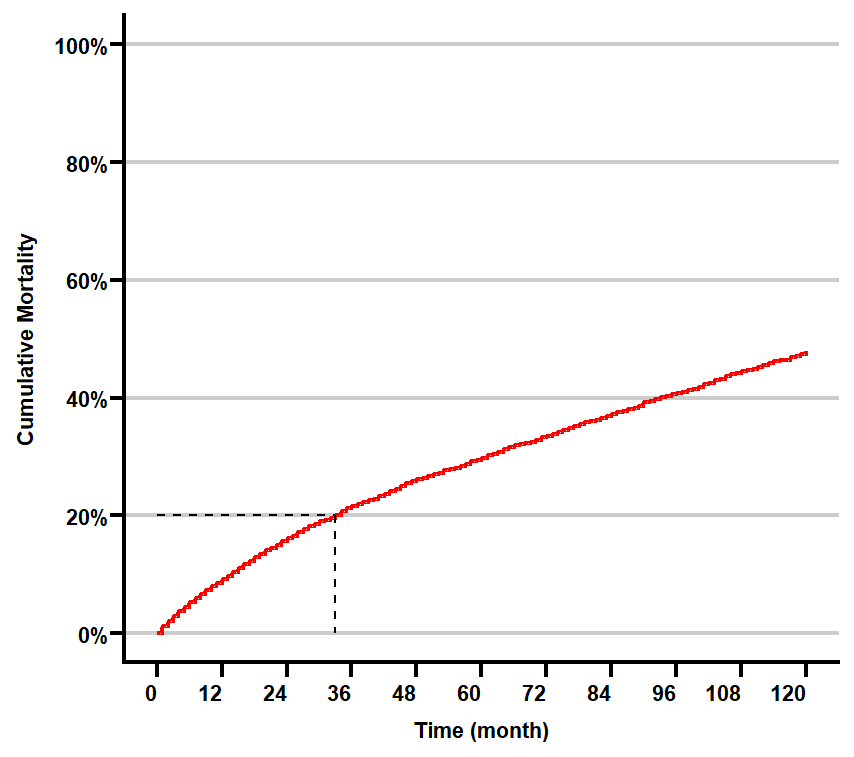


A

B

Supplementary Figure 5. Cumulative mortality curve of patients with thymic epithelial tumors


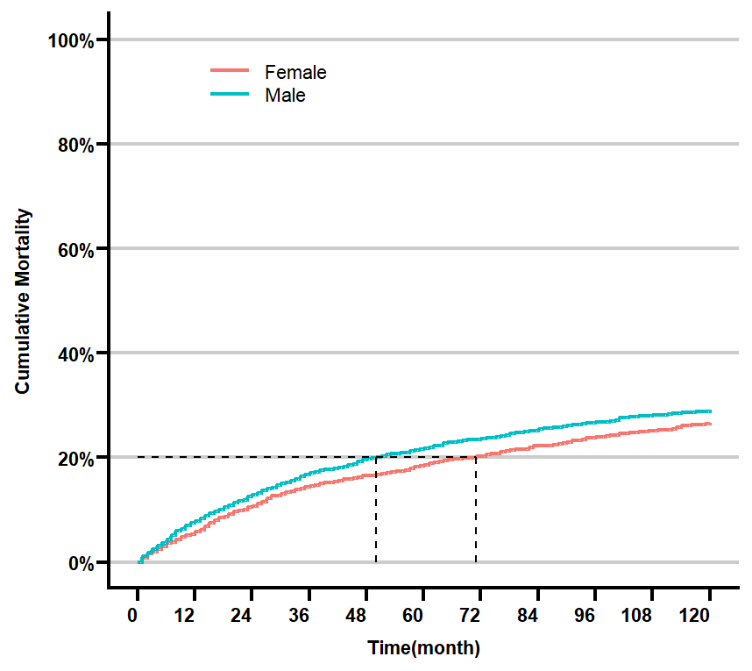


A


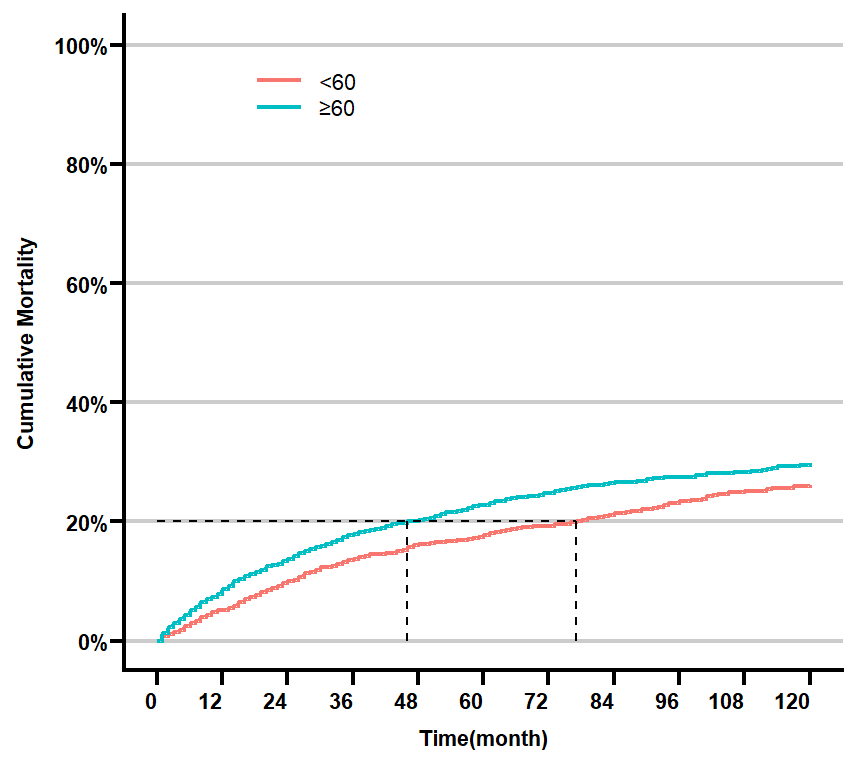


B

C


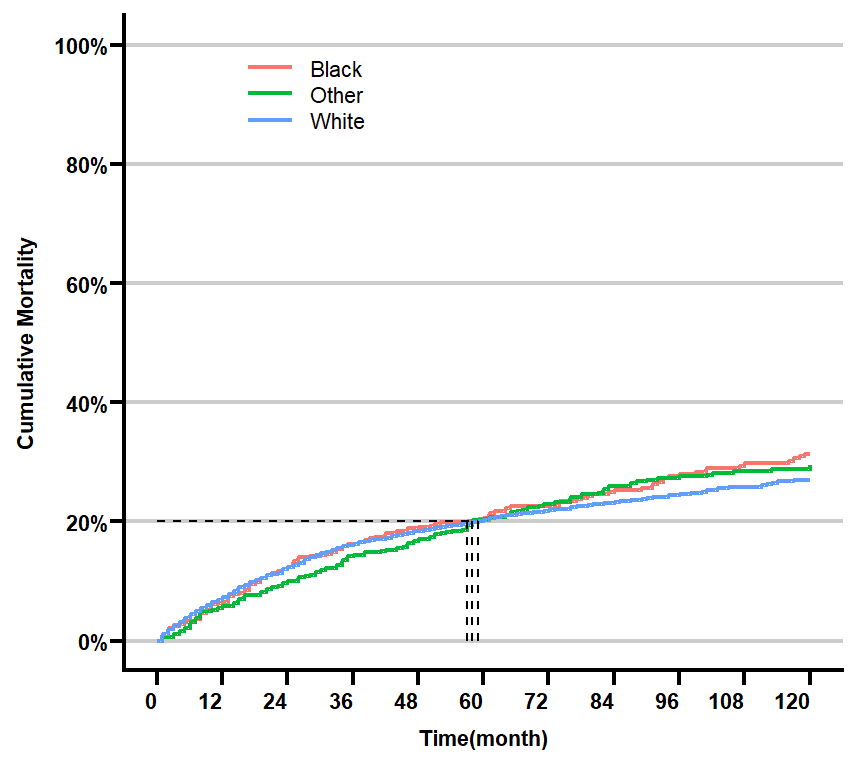


D


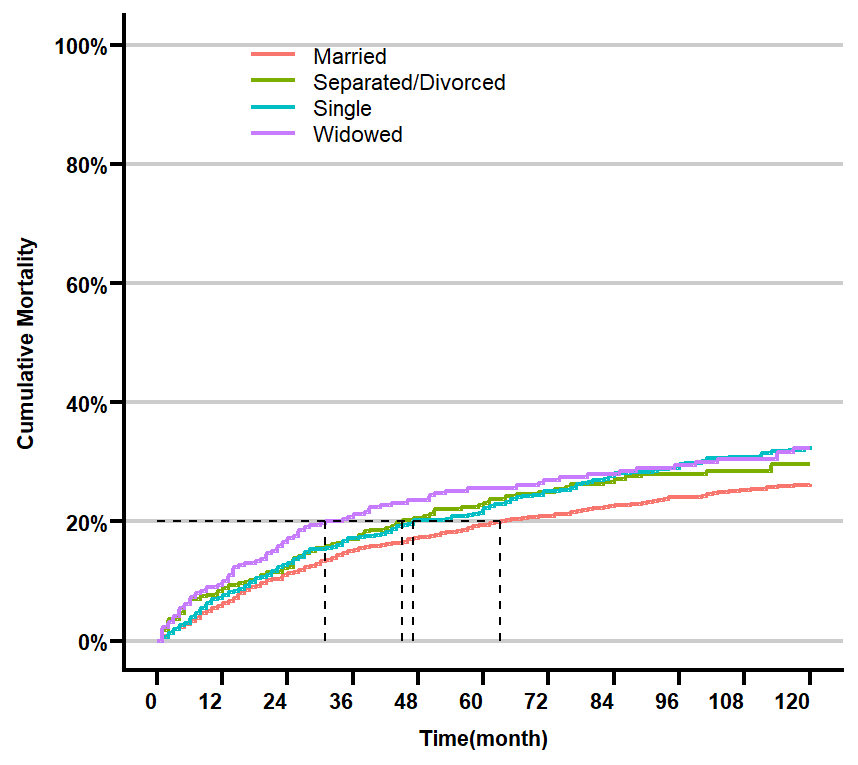


*P=0.015*

*P=0.090*

G

H

I

J


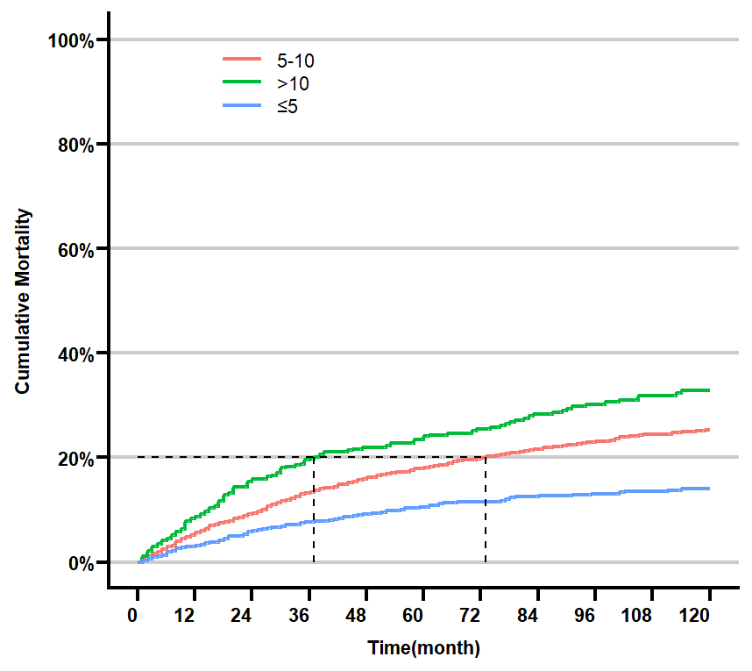

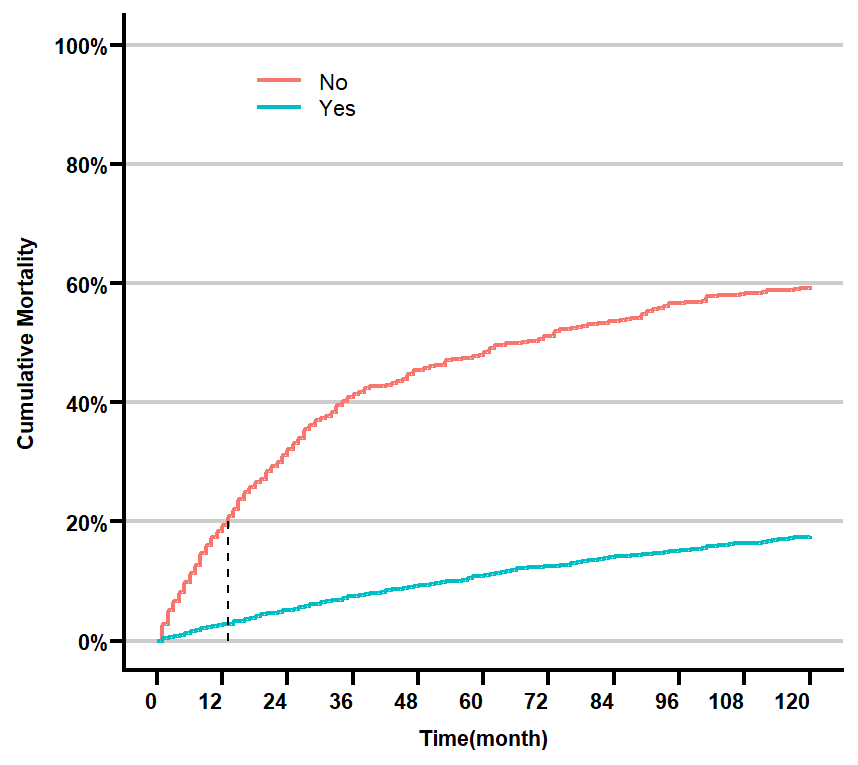

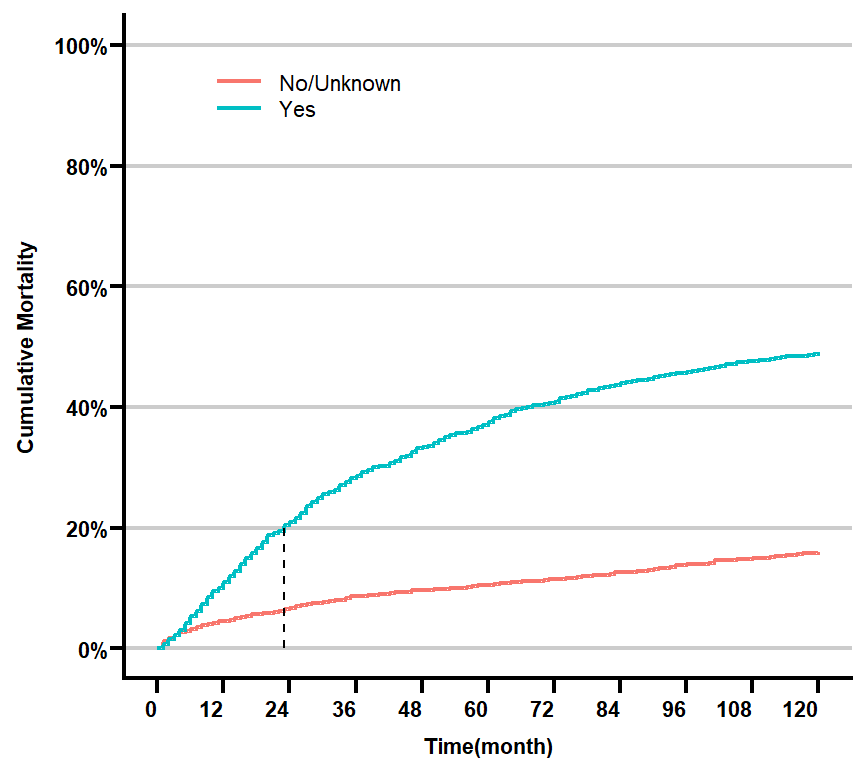

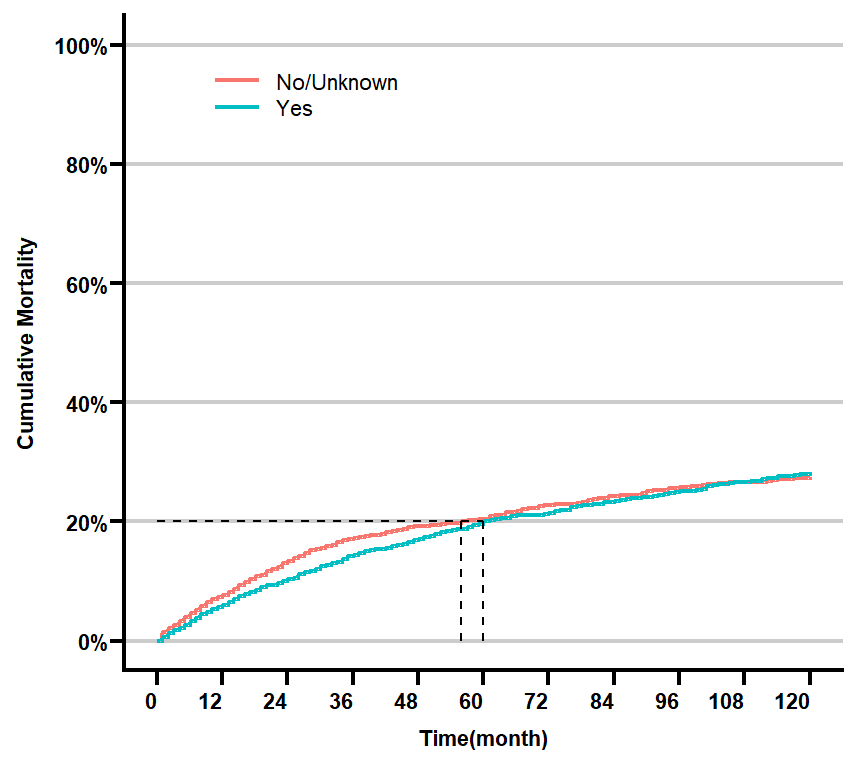


*P<0.001*

*P<0.001*

*P=0.560*


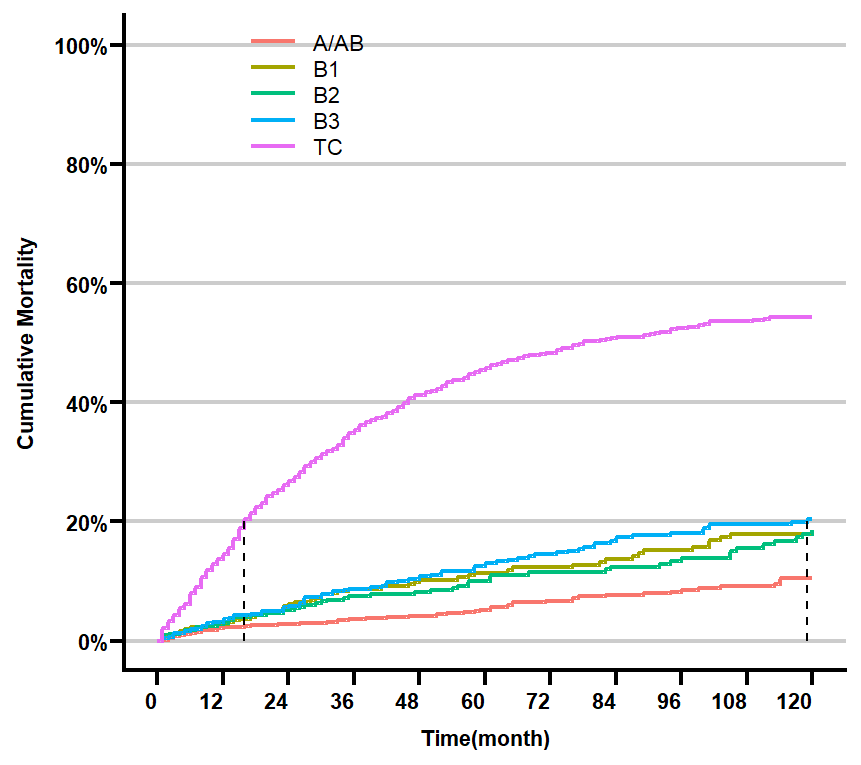


E


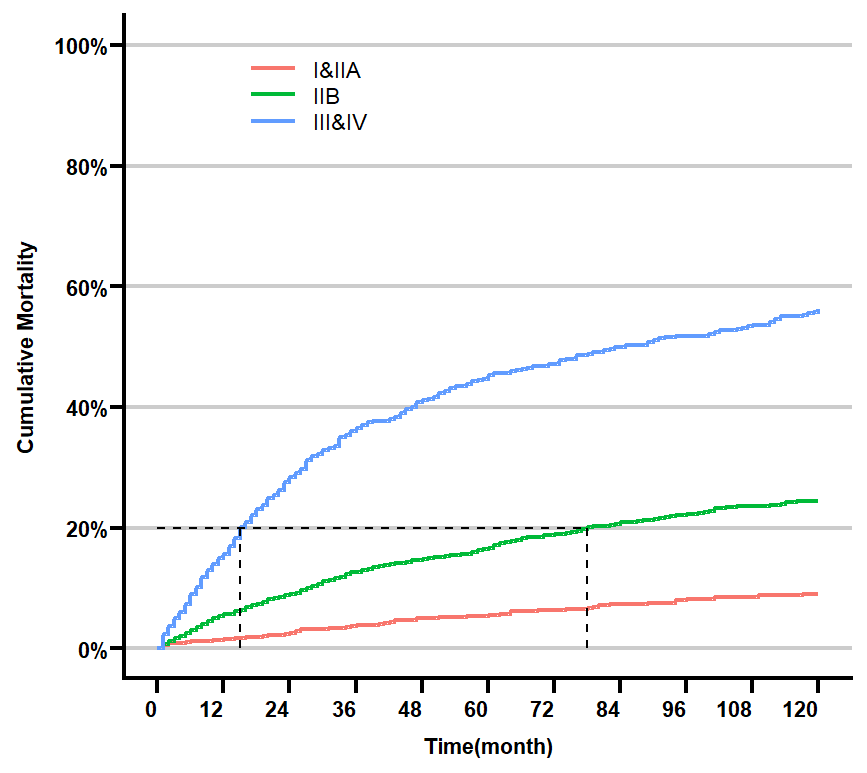


F

*P<0.001*

*P<0.001*

*P<0.001*

Supplementary Figure 6. Analysis of single factor competing risk model before multiple imputation of thymic epithelial tumor
